# Generation of cell type-specific monoclonal antibodies for the planarian and optimization of sample processing for immunolabeling

**DOI:** 10.1186/s12861-014-0045-6

**Published:** 2014-12-21

**Authors:** David J Forsthoefel, Forrest A Waters, Phillip A Newmark

**Affiliations:** Howard Hughes Medical Institute, Department of Cell and Developmental Biology, University of Illinois at Urbana-Champaign, B107 Chemical and Life Sciences Laboratory, 601 S. Goodwin Ave., Urbana, IL 61801 USA

**Keywords:** Planarian, Regeneration, Intestine, Monoclonal antibody screen, Immunohistochemistry, Immunofluorescence

## Abstract

**Background:**

Efforts to elucidate the cellular and molecular mechanisms of regeneration have required the application of methods to detect specific cell types and tissues in a growing cohort of experimental animal models. For example, in the planarian *Schmidtea mediterranea*, substantial improvements to nucleic acid hybridization and electron microscopy protocols have facilitated the visualization of regenerative events at the cellular level. By contrast, immunological resources have been slower to emerge. Specifically, the repertoire of antibodies recognizing planarian antigens remains limited, and a more systematic approach is needed to evaluate the effects of processing steps required during sample preparation for immunolabeling.

**Results:**

To address these issues and to facilitate studies of planarian digestive system regeneration, we conducted a monoclonal antibody (mAb) screen using phagocytic intestinal cells purified from the digestive tracts of living planarians as immunogens. This approach yielded ten antibodies that recognized intestinal epitopes, as well as markers for the central nervous system, musculature, secretory cells, and epidermis. In order to improve signal intensity and reduce non-specific background for a subset of mAbs, we evaluated the effects of fixation and other steps during sample processing. We found that fixative choice, treatments to remove mucus and bleach pigment, as well as methods for tissue permeabilization and antigen retrieval profoundly influenced labeling by individual antibodies. These experiments led to the development of a step-by-step workflow for determining optimal specimen preparation for labeling whole planarians as well as unbleached histological sections.

**Conclusions:**

We generated a collection of monoclonal antibodies recognizing the planarian intestine and other tissues; these antibodies will facilitate studies of planarian tissue morphogenesis. We also developed a protocol for optimizing specimen processing that will accelerate future efforts to generate planarian-specific antibodies, and to extend functional genetic studies of regeneration to post-transcriptional aspects of gene expression, such as protein localization or modification. Our efforts demonstrate the importance of systematically testing multiple approaches to species-specific idiosyncracies, such as mucus removal and pigment bleaching, and may serve as a template for the development of immunological resources in other emerging model organisms.

**Electronic supplementary material:**

The online version of this article (doi:10.1186/s12861-014-0045-6) contains supplementary material, which is available to authorized users.

## Background

Interest in the cellular and molecular mechanisms of regeneration has stimulated a resurgence of investigations in a growing cohort of organisms [1-4]. For example, planarians (freshwater flatworms) can recover from nearly any plane of transection, re-establishing their body plan and rebuilding internal organs such as the nervous and digestive systems within a week after injury [5-7]. Recent investigations have illuminated some of the mechanisms of planarian regeneration, including the re-establishment of axial polarity [8-10], somatic stem cell dynamics [11-15], tissue remodeling [16,17], organogenesis [17-24], reproductive maturation and germ cell development [25-28], and the molecular nature of regenerative competence [29-31]. As in many experimental animal models [32], these advances have required the adaptation and development of a range of tools and techniques, including methods for visualizing specific organs, tissues, and cell types. In particular, optimization of protocols for in situ hybridization [33-35] and sample processing for electron microscopy [16,21,36] have dramatically increased the resolution of regenerative events at the cellular level. By contrast, a rigorous analysis of the influence of specific steps during sample preparation for immunofluorescence has not been undertaken, and the collection of planarian-specific antibodies remains limited. Development of more systematic approaches for testing the effects of specific parameters on immunolabeling by individual antibodies would accelerate the generation of cell- and tissue-specific reagents, and expedite studies of post-transcriptional aspects of gene expression during regeneration, such as protein localization and modification.

Historically, characterization of planarian tissues and studies of their responses to injury were conducted using histological stains, vital dyes, and electron microscopy [37-40]. More recently, significant improvements to in situ hybridization (ISH) protocols [33-35] have enabled the use of RNA probes to label organs, subpopulations of cells, and ribonucleoprotein particles [19,22,26,27,41-47]. In addition to these methods, protocols utilizing both lectins and antibodies as cell-specific probes have also been developed. These protocols are less labor intensive and more economical than ISH protocols, and, in addition to detection of specific cell types, enable resolution of subcellular regions such as membranes, nuclei, and neuronal processes. A variety of lectins and antibodies (both monoclonal and polyclonal) have been generated or identified that label the secretory system [48], reproductive system [45], nervous system [49-55], intestine [52,56,57], protonephridia [21,22], muscles [17,58,59], and stem cells [11,56,60]. In addition, commercial and publicly available antibodies that cross-react with planarian antigens in the nervous system and other tissues have been identified [61-63].

Although antibodies are most often raised against specific molecules [64,65], monoclonal antibodies (mAbs) have also been generated in large-scale screens using purified cells, tissues, or whole-animal homogenates as immunogens. Such screens have yielded specific markers for neurons and their projections [66-74], regenerating tissues [75,76], and other cell types [77-79] in a variety of organisms. Tissue-based mAb screens bypass potential difficulties such as the need to identify highly expressed proteins or immunogenic regions appropriate for production of fusion proteins. Additionally, mAb screens result in the generation of clonal, immortal hybridoma lines and therefore a theoretically inexhaustible supply of antibody [80]. Several mAb screens have been conducted using planarian cells and extracts, and have yielded markers for various tissues and cell types [58,81-85].

Despite recent progress, the repertoire of antibodies that recognize planarian tissues is still limited, and a greater collection of reagents that label cell types unique to specific organ systems is needed. For example, regeneration of the intestine requires both remodeling of pre-existing, post-mitotic intestinal tissue and addition of new intestinal cells at the growing ends of regenerating gut branches [17]. Antibodies currently used to label the intestine [52,56,57] lack specificity, labeling additional tissues such as pharynx, epidermis, and nervous system. Furthermore, some of these antibodies label only subregions of intestinal cells such as the apical surface, making them less than ideal for analysis of remodeling and growth during intestinal morphogenesis.

In order to develop more specific intestinal antibodies, we took advantage of a protocol we recently developed [23] that enables purification of intestinal cells, and conducted a mAb screen using these purified cells as immunogens. We generated ten mAbs that labeled the intestine, as well as 13 mAbs that label the nervous system, epidermis, secretory cells, and other cell types. Because sample processing is known to influence antibody-antigen interactions [86-89], we also systematically evaluated the effects of various parameters during fixation of planarian tissue, including chemical treatments to remove mucus and pigmentation. This analysis led to the identification of optimal sample preparation protocols for several mAbs, and to the development of an optimization workflow that efficiently tests the influence of multiple processing steps on immunolabeling in planarians.

## Results and discussion

### A monoclonal antibody screen utilizing purified intestinal phagocytes as immunogens

**Figure 1:**
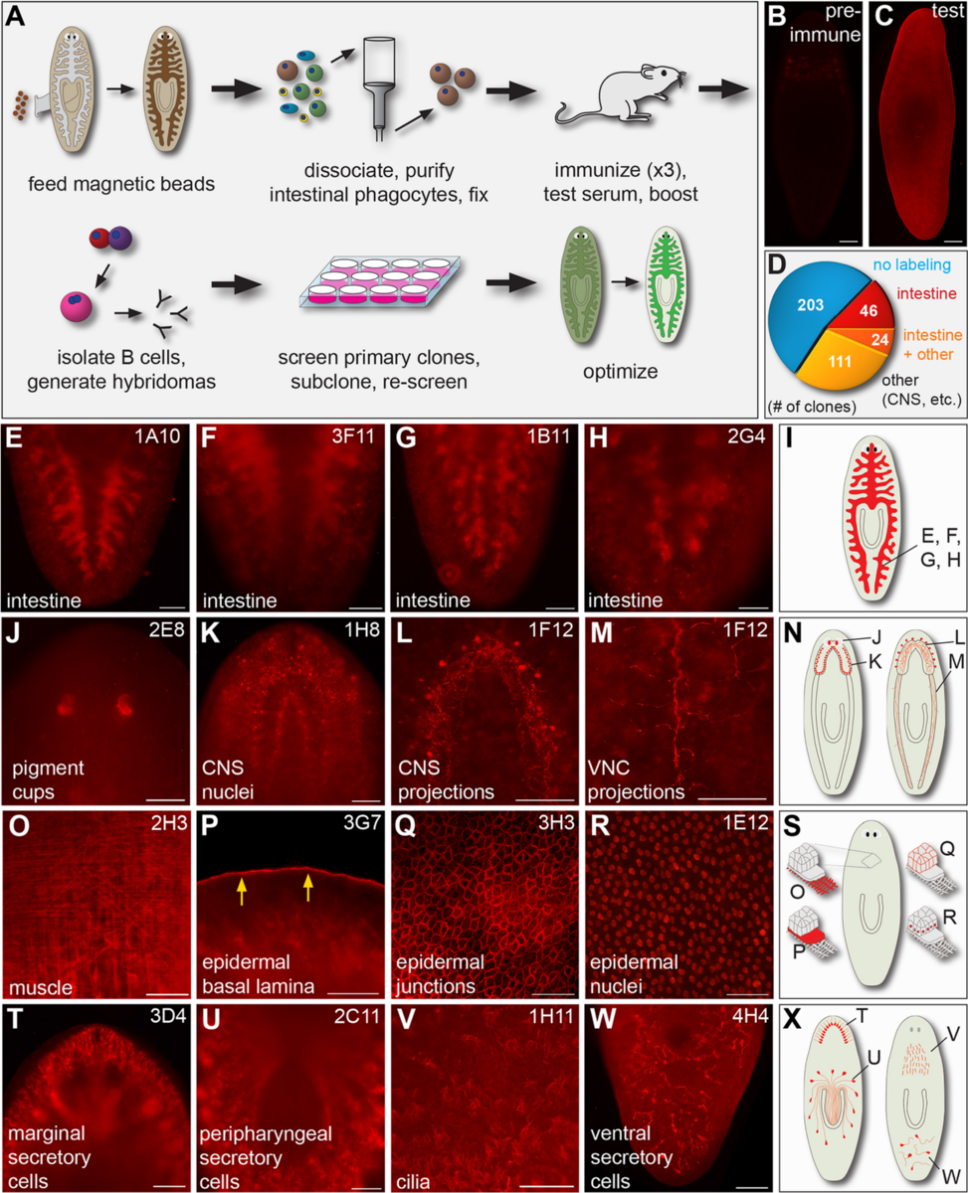
**Generation of monoclonal antibodies that recognize the planarian intestine and other tissues. (A)** Screen strategy. **(B)** Preimmunization serum (1:200) did not detectably label planarian tissue. **(C)** Test sera (1:200) ubiquitously labeled planarian tissues, with slightly elevated intestinal labeling. **(D)** Pie chart depicting the number of clones (n = 384 total) in the primary screen with indicated labeling specificities. **(E-X)** Examples and schematics of immunolabeling from the primary screen. **(E-I)** mAbs that label the intestine (schematic, **I**). **(J-N)** mAbs that label the nervous system (schematic, **N**). **(O-X)** Examples of mAbs that label other non-intestinal tissues (schematics, **S** and **X**). Anterior is to the top in all images. Cell and tissue types are indicated in each panel. “CNS”, central nervous system. “VNC”, ventral nerve cord. Scale bars: 100 μm **(B, C, E-H, J-M, O, T, U,** and **W)**; 50 μm **(P-R)**; 25 μm **(V)**.

The planarian intestinal epithelium is comprised of two cell types: secretory goblet cells that release digestive enzymes after food ingestion, and absorptive phagocytes that engulf food for intracellular digestion [90-92]. Intestinal phagocytes retain dyes, beads, and other compounds for up to several weeks after they are ingested [17,93-96]. We recently developed a protocol in which animals are fed iron beads and dissociated into single cell suspensions, enabling the purification of phagocytes by magnetic sorting (Figure 1A) [23]. Using this protocol (Additional file 1), we collected phagocytes from more than 4000 planarians. After fixation, these cells were used as immunogens for a monoclonal antibody screen.

Three mice were immunized; test bleeds from all three mice labeled fixed planarians, while preimmune sera did not (examples from one mouse are shown in Figure 1B and C). Immunofluorescence was ubiquitous, likely reflecting the fact that many intestinal antigens are also expressed in other planarian cell types (Figure 1C). We also observed slightly elevated signal in the intestine for all three test sera (Figure 1C). One mouse was chosen for final immunization (“boost”) and hybridoma generation.

After fusion of splenocytes with myeloma cells and cloning of the resulting hybridomas, supernatants from 384 primary lines were screened for immunoreactivity with fixed planarians (Figure 1D-X). In total, 181 (~47%) supernatants labeled planarian tissues at levels visibly higher than secondary antibody controls (Figure 1D). Seventy (~22%) supernatants labeled the intestine (Figure 1E-I). In addition, 135 supernatants (~35%) labeled non-intestinal tissues (Figure 1J-X). These included pigment cells of the optic cup (Figure 1J), neurons and their processes (Figure 1K-M), muscles (Figure 1O), epidermis (Figure 1P-R), secretory cells (Figure 1T, U and W) (with morphologies and locations similar to previously described cells [48,61]), and cilia (Figure 1V). Twenty-four (~6%) antibodies that labeled non-intestinal tissues also labeled the intestine (e.g., Figure 1T), suggesting the recognition of highly immunogenic antigens that are expressed at lower levels in the intestine than in other tissues. Alternatively, some non-intestinal antibodies might also have been generated against antigens expressed by contaminating cells or cell fragments in intestinal cell preparations. In previous monoclonal screens, less than 8% of antibody-producing clones labeled intestinal tissue [58,81]. By contrast, in our screen ~25% of positive clones (46/181) labeled the intestine with marked specificity. Because intestinal cells comprise only 3-8% of all planarian cell types [97], these results show that immunization with an enriched cell population is an efficient strategy for generating cell type-specific mAbs.

We retained 23 hybridomas, giving preference to those that produced antibodies labeling the intestine and other specific cell types with high signal and low background (Table 1). 21 of 23 hybridoma lines produced IgM class antibodies, while two lines produced IgG mAbs (Table 1). IgM-producing hybridomas can dominate an immune response due to underimmunization with suboptimal amounts of antigen, or an abundance of complex carbohydrates and lipids when whole cells are used as an immunogen [65,98,99]. Consistently, a recent effort to generate mAbs against membranes purified from planarian stem cells yielded many IgM-producing lines [85]. Although IgM mAbs sometimes have lower affinity, reduced specificity, and decreased ability to penetrate tissue [98,99], our screening strategy identified IgM mAbs that labeled specific cell types and penetrated tissues well. Each of the 23 hybridomas was subcloned, and we identified at least one unique line that continued to secrete high levels of specific mAb after rescreening, freezing, and expansion. Subclones were deposited in the Developmental Studies Hybridoma Bank (Iowa City, IA) for archival and public distribution.

**Table 1:** **Monoclonal antibodies**, **isotypes**, **and tissue specificity**

**Clone**	Isotype*	**Tissue(s) recognized**
1A10	IgM	Intestine
1B11	IgM	Intestine^†^
1C3	IgM	Intestine^†^
1E12	IgM	Epidermal & intestinal nuclei
1F12	IgM	CNS neurons (small subset)
		CNS processes in neuropil (subset)
		PNS processes in neuropil (subset)
1H8	IgM	Nuclei (elevated in CNS)
1H11	IgM	Cilia^†^
2C11	IgG1	Peripharyngeal secretory cells & projections
2D2	IgM	CNS neurons (small subset)
		CNS processes in neuropil (subset)
		PNS processes in neuropil (subset)
2E8	IgM	Pigment cups^†^
2G4	IgM	Intestine & epidermis
2H3	IgM	Muscles
3A4	IgM	Intestine^†^
3A10	IgM	Intestine^†^
3D4	IgM	Marginal/anterior secretory cells intestine (weak)^†^
3D10	IgM	Ventral puncta^†^
3F11	IgG1	Intestine
3G7	IgM	Epidermal basement membrane
3G9	IgM	Intestine
3H3	IgM	Epidermis, enriched in cell:cell junctions
4D2	IgM	Intestine
4H4	IgM	Ventral secretory cells
4H8	IgM	Epidermal nuclei (subset)^†^

*All mAbs possessed kappa light chains.

^†^Pattern based on primary screen; mAb has not been characterized further.

### Optimization of planarian relaxation, mucus removal, and fixation improves immunolabeling

Although our mAbs consistently labeled specific tissues in the primary screen, levels of both signal and non-specific background often varied from animal to animal and within different regions of individual animals. Because epitope accessibility and antibody specificity depend critically on fixative choice and other sample processing steps [87,88,100-104], we hypothesized that optimization of fixation and other treatments would improve labeling with some mAbs.

**Figure 2:**
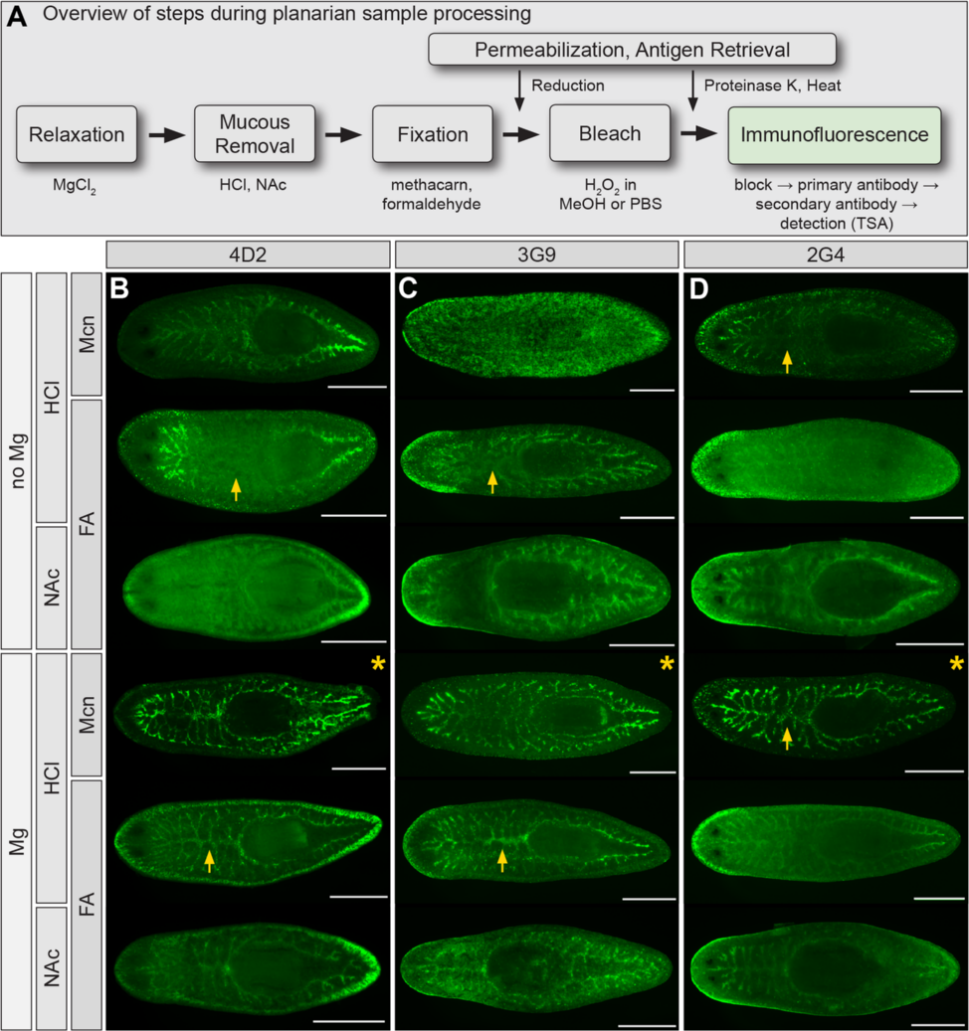
**Sample processing influences immunofluorescent labeling of the planarian intestine. (A)** Overview of steps during fixation and processing. **(B)** mAb 4D2 labeling. **(C)** mAb 3G9. **(D)** mAb 2G4. For all three mAbs, labeling was best (i.e., highest signal and lowest non-specific background) in animals relaxed in magnesium and fixed with methacarn (asterisks). In some samples, magnesium treatment also improved labeling of anterior intestinal branches (arrows). “Mg”, magnesium-induced relaxation. “NAc”, N-Acetyl-L-cysteine treatment. “HCl”, HCl treatment. “FA”, formaldehyde/Triton X-100 fixation. “Mcn”, methacarn fixation. “TSA”, tyramide signal amplification. Samples were bleached in methanol/6% H_2_O_2_ (16-20h). Scale bars: 500 μm.

Processing of planarian tissue for immunofluorescence requires several steps in addition to fixation, including chemical removal of mucus secretions prior to fixation, bleaching to remove pigmentation and enable visualization of internal structures, and post-fixation treatments to increase tissue permeability and expose antigens (Figure 2A). A range of approaches to these steps have been developed to prepare planarians for whole mount in situ hybridization, immunolabeling, and histological analysis [11,17,33-35,49,90,105-108]. In our primary screen, we tested hybridoma supernatants on HCl-treated, formaldehyde-fixed planarians that were bleached in hydrogen peroxide diluted in PBS. Because formal demonstrations of the effects of specific treatments at each stage of sample processing are rarely reported, we evaluated additional approaches to these steps simultaneously by testing combinations of treatments together (detailed protocols are provided in Additional file 2).

We began by comparing two chemical treatments commonly used to relax planarians and remove their mucus secretions prior to fixation: hydrochloric acid (HCl) [33] and N-Acetyl-L-cysteine (NAc) [34]. We also compared formaldehyde, a cross-linking fixative [81,82,107], with methacarn, a coagulating fixative [17,45], reasoning that antibodies raised against cells fixed in formaldehyde and methanol would be most likely to react with planarian tissues that had been fixed similarly. Additionally, we tested magnesium chloride, which has been used to relax marine and freshwater invertebrates prior to fixation [109,110], as an extra step prior to mucus removal. We have previously utilized magnesium-induced relaxation to increase resolution of individual branches of the planarian intestine [23]. For these optimizations, we utilized indirect detection (tyramide signal amplification, TSA), which dramatically improved signal intensity for supernatants generated after the primary screen (Additional file 3: A-F). Furthermore, blocking time was increased to overnight (15-18 hr), a step that moderately improved signal-to-noise for some intestinal antibodies (Additional file 3: G and J), without affecting labeling efficiency for others (Additional file 3: H, I, K and L).

For intestine-specific mAbs, magnesium treatment universally improved labeling (Figure 2B-D). For example, in the absence of magnesium treatment, mAbs 4D2 and 3G9 labeled anterior and posterior intestinal branches in HCl-treated, formaldehyde-fixed planarians, but penetration in the prepharyngeal region was poor (Figure 2B and C). When animals were relaxed in magnesium prior to HCl treatment, labeling of the primary anterior intestinal branch was dramatically improved. Similar results were observed for a third mAb, 2G4, in methacarn-fixed planarians (Figure 2D).

Mucus removal method also affected intestinal labeling, albeit more moderately. For example, HCl treatment improved signal and reduced non-specific labeling by antibodies 4D2 and 3G9 (Figure 2B and C) in formaldehyde-fixed planarians, while NAc treatment led to slightly more specific labeling by 2G4 (Figure 2D). We also attempted to fix NAc-treated animals with methacarn, but animals disintegrated during bleaching (not shown). Milder NAc treatments or reduced H_2_O_2_ concentration during bleaching improved integrity, but abolished intestinal labeling (not shown). Although we cannot rule out that some antibodies might work well on such samples, we did not test NAc-methacarn combinations further in whole planarians.

Fixative choice was also critical for efficient immunofluorescence. For most intestine-specific antibodies we tested (4D2, 3G9, and 2G4), methacarn fixation was superior to formaldehyde fixation, especially in Mg-treated animals (Figure 2B-D). In methacarn-fixed specimens, intestinal labeling was more intense, particularly in anterior regions. Additionally, non-specific background labeling was much lower as compared to formaldehyde-fixed planarians. These results show that processing conditions (e.g., formaldehyde fixation) utilized to screen hybridomas do not automatically identify the “best” treatment conditions for particular antibodies.

**Figure 3:**
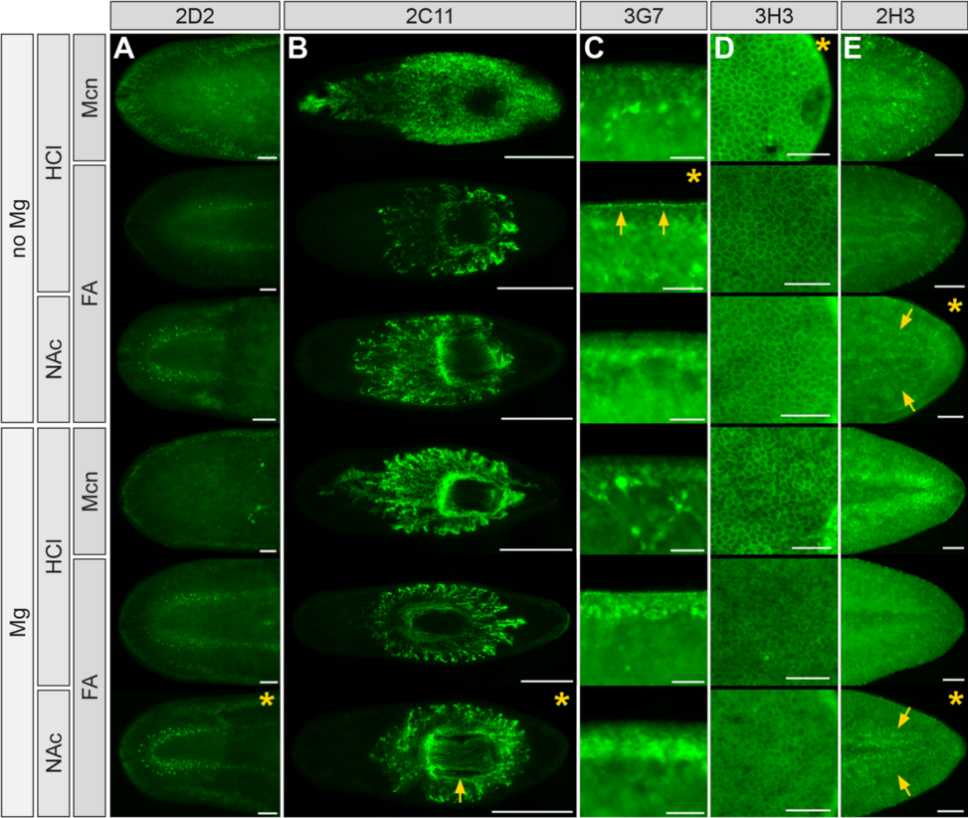
**Effects of fixation and other parameters on immunofluorescent labeling of non**-**intestinal tissues. (A)** mAb 2D2 labels a subset of CNS neurons and processes after formaldehyde fixation. **(B)** mAb 2C11 labels cell bodies around the pharynx as well as fine processes within the pharynx (arrow). **(C)** mAb 3G7 labeling of the epidermal basement membrane (arrows) is negatively affected by magnesium treatment, methacarn fixation, and NAc treatment. **(D)** mAb 3H3 labeling of epidermal cell:cell junctions. Images taken from the posterior of the animal. Region shown is the lateral margin of the animal adjacent to the pharynx. **(E)** mAb 2H3 labeling of body wall and enteric musculature (arrows). Sample treatment parameters are indicated at left. In all panels, anterior is to the left. Yellow asterisks indicate conditions that yielded the most specific signal with minimal background labeling. All samples were bleached in methanol (16-20 hr). Scale bars: 100 μm **(A**, **E)**; 500 μm **(B)**; 50 μm **(C**, **D)**.

Non-intestinal mAbs displayed a similar range of sensitivity to combinations of treatments (Figure 3A-E; Additional file 4: A and B). For example, in formaldehyde-fixed samples, mAb 2D2 labeled a subset of neuronal processes in the cephalic ganglia and ventral nerve cords, as well as a small number of cell bodies anterior and lateral to the brain (Figure 3A; Additional file 3: E, H, and K). Labeling was enhanced by both Mg and NAc treatments, but abolished by methacarn fixation. Similarly, mAb 1H8 preferentially labeled CNS nuclei in NAc-treated, formaldehyde-fixed samples, and also in Mg-treated, formaldehyde-fixed samples, whether HCl or NAc was used for mucus removal (Additional file 4: A). mAb 2C11 (specific for peripharyngeal secretory cells and their processes [61]) labeled efficiently in both methacarn and formaldehyde (Figure 3B). Labeling of processes in the pharynx, a more internal tissue, was improved in Mg-, NAc-treated samples (Figure 3B). By contrast, Mg and NAc treatment reduced or eliminated labeling by mAb 3G7, which labeled the epidermal basement membrane only in HCl-treated, formaldehyde-fixed animals (Figure 3C). Similarly, labeling of epidermal cell junctions by mAb 3H3 (Figure 3D) was also negatively affected by Mg treatment, suggesting that magnesium may improve penetration of some mAbs at least in part through mild histolysis of more superficial tissues. Finally, mAb 2H3 labeled superficial body wall muscles best in NAc treated samples, while labeling of internal enteric muscles surrounding intestinal branches was less sensitive to sample preparation (Figure 3E).

Taken together, these initial optimizations demonstrate that epitopes recognized by our collection of mAbs are selectively sensitive to combinations of commonly used mucus removal treatments and fixatives (Table 2). Furthermore, magnesium-induced relaxation is a simple step that can improve labeling of deeper tissues such as the intestine and pharynx. Optimization of these steps together is an efficient method for improving signal and reducing non-specific background labeling.

**Table 2:** **Optimization of whole animal sample processing**

**mAb**	**Tissue specificity**	**Fixation protocol**					
					**Mg**	**Mg**	**Mg**
		**HCl**	**HCl**	**NAc**	**HCl**	**HCl**	**NAc**
		**Mcn**	**FA**	**FA**	**Mcn**	**FA**	**FA**
2G4	Intestine & epidermis	+	+	+	++	+	+
3F11	None (MeOH bleach)	-	-	-	-	-	-
3F11	Intestine (PBS bleach)	-	-	-	-	+	+
3G9	Intestine	-	+	+	++	+	+
4D2	Intestine	+	-	-	++	+	+
1E12	Nuclei, elevated in epidermis and intestine	+	+	-	+	-	-
1H8	Nuclei, elevated in CNS	-	-	+	-	+	+
2C11	Peripharyngeal secretory cells and processes	-	+	+	-	+	++
2D2	Subset of neurons and processes	-	+	++	-	+	++
2H3	Muscles	++	+	++	++	+	++
3G7	Epidermal basement membrane	-	+	-	-	+	-
3H3	Epidermis, enriched in cell:cell junctions	++	+	+	++	-	-

Qualitative assessment of signal:noise for the predominant tissue labeled by each mAb. “-“ indicates negligible or non-uniform labeling, poor morphology, and/or high background. “+“ indicates moderate specificity, uniform labeling, and/or robust labeling accompanied by moderate background. “++” indicates robust signal with minimal noise.

### Further optimization of hydrogen peroxide treatment and other sample processing steps

For many mAbs, identification of the appropriate combination of magnesium treatment, mucus removal method, and fixative was sufficient to yield reproducible and specific labeling. For several mAbs, however, labeling was still inconsistent and accompanied by higher levels of background, particularly in anterior regions where secretory cells are more abundant [48]. In an effort to further improve labeling, we conducted more detailed analyses of bleaching and mucus removal steps, and also tested the effects of post-fixation treatments to unmask epitopes and permeabilize tissue. We chose three of the most inconsistent mAbs (3F11, 2D2, and 2C11) that labeled different cell types from both superficial and internal tissues for further analysis of these steps.

**Figure 4:**
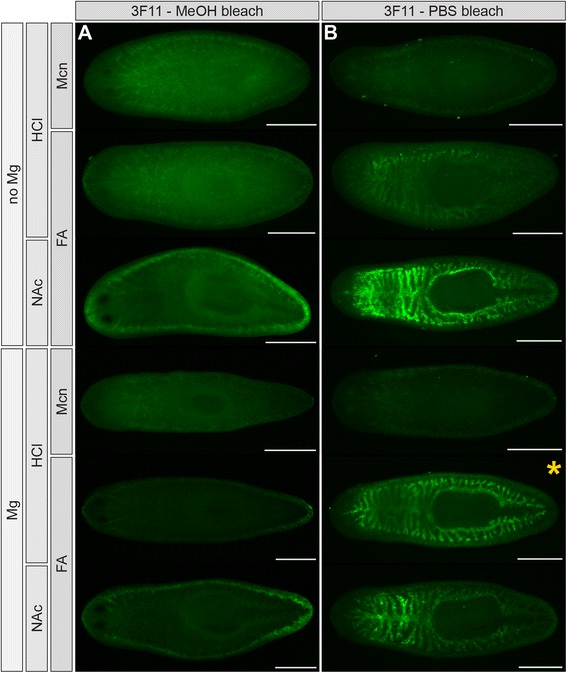
**Methanol bleaching abolishes labeling by mAb 3F11. (A)** mAb 3F11 does not label samples bleached in methanol (12-16 hr). **(B)** mAb 3F11 labels the intestine in formaldehyde-fixed animals bleached in PBS (12-16 hr). Mg treatment also substantially improves 3F11 labeling in HCl-treated samples, but substantially reduces labeling in NAc-treated samples **(B)**, demonstrating that some antibodies are uniquely sensitive to specific combinations of treatments. In all panels, anterior is to the left. Scale bars: 500 μm.

In order to facilitate visualization of internal tissues, hydrogen peroxide bleaching prior to immunolabeling has become a routine post-fixation step [49]. Bleaching also likely increases tissue permeability, allowing better penetration of molecular probes [35]. In published protocols, hydrogen peroxide is usually diluted with PBS, methanol, or other diluents, but the degree to which labeling by individual antibodies is affected by diluent choice is rarely reported. In our primary screen (Figure 1), hydrogen peroxide was diluted with PBS. In subsequent optimizations (Figures 2 and 3), samples were bleached in hydrogen peroxide diluted with methanol. Although most antibodies were unaffected by bleaching diluent, methanol bleaching completely abolished intestinal labeling by one antibody, 3F11 (Figure 4A and B). Consistently, no intestinal labeling was observed in methacarn-fixed animals, even when bleached in PBS, suggesting that the epitope recognized by mAb 3F11 is sensitive to methanol (Figure 4B).

**Figure 5:**
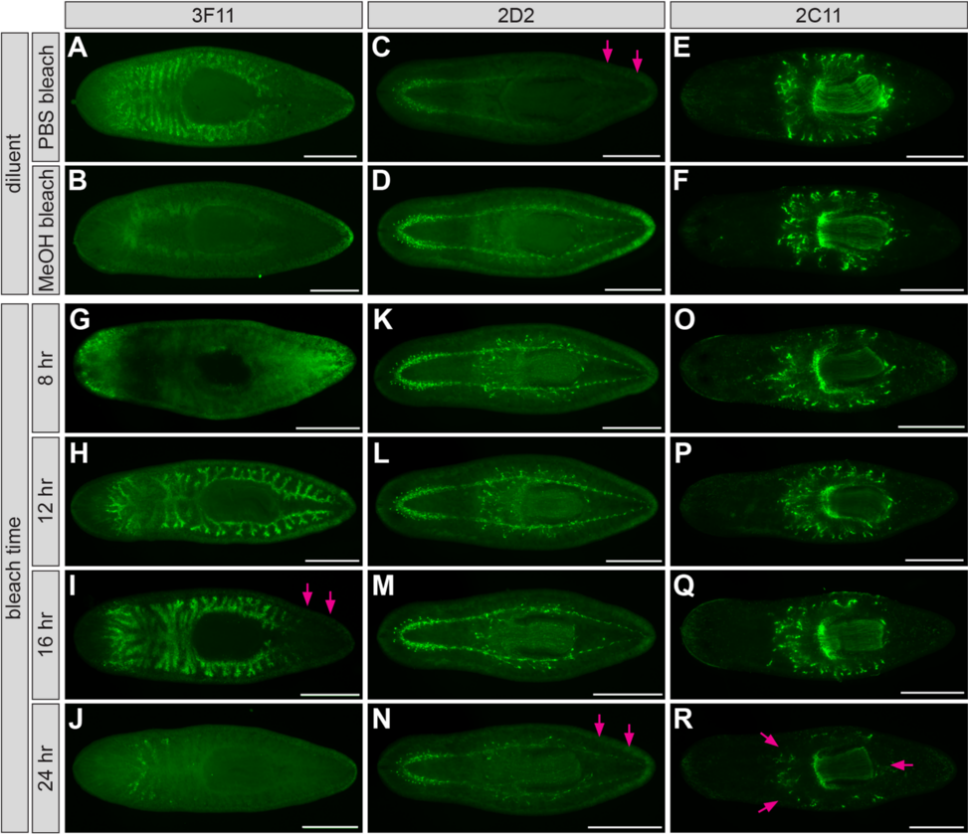
**Bleaching affects mAb labeling. (A**-**B)** Planarians labeled with mAb 3F11 after hydrogen peroxide bleaching in either PBS **(A)** or methanol **(B)**. Labeling is completely abolished by methanol bleaching. **(C**-**D)** mAb 2D2 labeling of the CNS is more robust in samples bleached in methanol, particularly in posterior regions (arrows indicated reduced signal in **C**). **(E**-**F)** mAb 2C11 labeling is unaffected by bleaching diluent. **(G**-**J)** Planarians labeled with mAb 3F11 after bleaching for the times indicated at left. Arrows **(I)** indicate signal loss in tail branches. **(K**-**N)** Planarians labeled with mAb 2D2. Arrows **(N)** indicate signal loss in tail branches. **(O**-**R)** Planarians labeled with mAb 2C11. Arrows **(R)** indicate decreased signal both within and around the pharynx. Planarians were relaxed in magnesium chloride, treated with 2% HCl (3F11) or 7.5% NAc (others), fixed in formaldehyde/Triton X-100, and bleached in 6% H_2_O_2_/PBS (3F11) or 6% H_2_O_2_/methanol (others). Samples were bleached for 12 hr in **A**-**F**. Scale bars: 500 μm.

To further explore diluent choice, we compared the effects of bleaching in PBS or methanol for two additional mAbs. Unlike 3F11 labeling (Figure 5A and B), 2D2 labeling of the CNS was significantly reduced in PBS-bleached animals, particularly in more posterior regions (Figure 5C and D). 2C11 labeling, on the other hand, was robust whether bleaching was carried out in either PBS or methanol (Figure 5E and F). Thus, although many of the mAbs we generated labeled planarians bleached in either PBS or methanol, side-by-side analysis indicates that bleaching diluent is an important variable that can influence optimal immunofluorescence.

Antibody labeling was also sensitive to the duration of bleaching. Many published protocols vaguely specify “overnight” bleaching, but systematic analysis of how bleaching time affects immunolabeling has not been reported. Again, most of our mAbs adequately labeled samples bleached overnight (14-16 hr in our protocols) (Figures 2 and 3; Additional file 4). However, 3F11 was sensitive to both over- and under-bleaching in both HCl-treated (Figure 5G-J) and NAc-treated (Additional file 5: A-D) planarians. In addition, labeling of posterior gut branches was specifically decreased in moderately overbleached samples (Figure 5I). Other mAbs (2D2, 2C11, and 3G9) were also sensitive to overbleaching to varying degrees (Figure 5K-R and Additional file 5: E-H). Antigen sensitivity to hydrogen peroxide treatment has been previously observed for a variety of samples from various organisms [99]. Our results show that although bleaching is essential for immunolabeling of whole planarians, duration of hydrogen peroxide treatment should be optimized for individual antibodies. Furthermore, caution is warranted when evaluating antibodies raised against proteins whose expression or localization is expected to vary along the anteroposterior axis.

**Figure 6:**
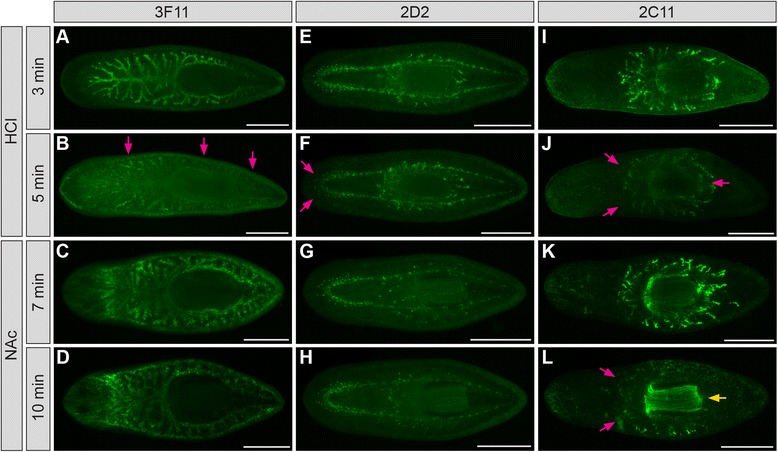
**Extended mucus removal time attenuates immunofluorescent labeling. (A-D)** Planarians labeled with mAb 3F11 after HCl **(A-B)** or NAc **(C-D)** treatment for the times indicated. **(E-H)** Planarians labeled with mAb 2D2. **(I-L)** Planarians labeled with mAb 2C11. Planarians were relaxed in magnesium chloride, treated with 2% HCl or 7.5% NAc, fixed in formaldehyde, and bleached in 6% H_2_O_2_/PBS (3F11) or 6% H_2_O_2_/MeOH (others) (12-16 hr). Magenta arrows indicate reduced labeling. Yellow arrow **(L)** indicates increased labeling of pharynx. Scale bars: 500 μm.

Next, we tested the effect of increasing the duration of mucus removal treatments. For mAb 3F11, longer HCl and NAc treatments both substantially reduced labeling (Figure 6A-D). For 2D2, longer HCl treatment moderately reduced labeling in the anterior of the animal (Figure 6E and F), while longer NAc treatment had no effect (Figure 6G and H). For 2C11, longer HCl treatment reduced labeling, as with 3F11 and 2D2 (Figure 6I and J). However, longer NAc treatment increased 2C11 labeling within the pharynx, while simultaneously reducing labeling of peripharyngeal cell bodies and projections (Figure 6K and L). Thus, longer mucus removal times generally reduce labeling intensity, but in some cases might benefit immunolabeling by increasing the accessibility of more internal organs like the pharynx.

**Figure 7:**
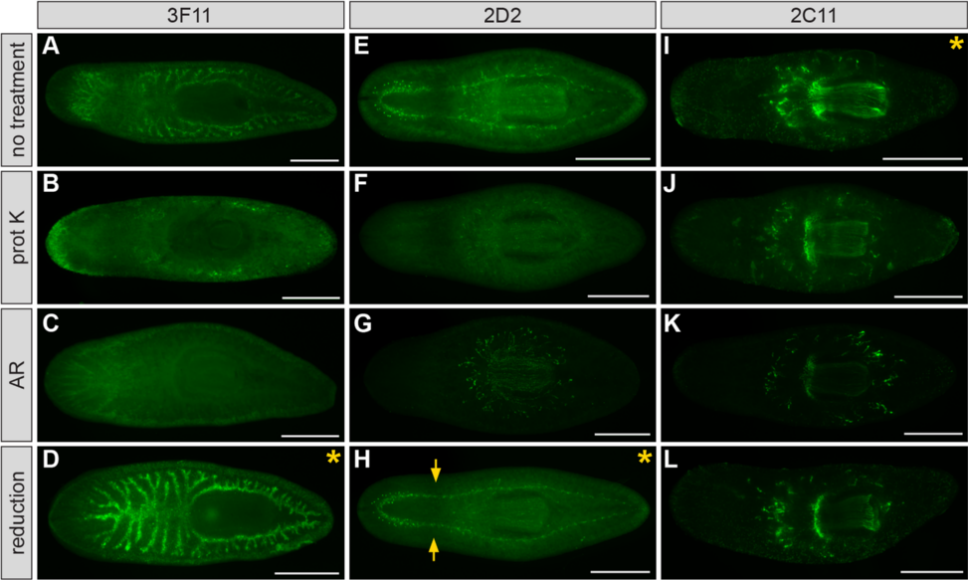
**Post**-**fixation treatments influence immunolabeling. (A**-**D)** Planarians labeled with mAb 3F11 after the treatments indicated at left. **(E**-**H)** Planarians labeled with mAb 2D2. **(I**-**L)** Planarians labeled with mAb 2C11. Arrows in **(H)** indicate the prepharyngeal region that is permeabilized by reduction treatment (compare to **E**). Asterisks indicate “best” treatment (i.e., highest signal intensity and specificity). Planarians were relaxed in magnesium chloride **(A-D)**, treated with 2% HCl **(A-D)** or 7.5% NAc **(E-L)**, fixed in formaldehyde/Triton X-100, and bleached in 6% H_2_O_2_/PBS **(A-D)** or 6% H_2_O_2_/MeOH **(E-L)** (12-16 hr). Reduction was conducted prior to bleaching, while proteinase K (“prot K”) and antigen retrieval (“AR”) were conducted after bleaching. Scale bars: 500 μm.

Finally, we hypothesized that treatments to increase tissue permeability and “unmask” antigens might improve mAb labeling. We tested three methods commonly used on whole planarians: reduction, which chemically permeabilizes tissues [44,111]; proteinase K treatment, which is thought to enzymatically cleave fixation-induced bonds [34,112,113]; and heat-induced antigen retrieval (“AR”), which increases antigenicity for some epitopes by a mechanism that remains incompletely understood [17,35,114,115] (Figure 7). Proteinase K digestion and AR were detrimental, reducing or completely abolishing labeling for 3F11, 2D2, and 2C11 (Figure 7A-C, E-G, and I-K). Reduction, on the other hand, improved labeling for 3F11 and 2D2, increasing antibody penetration in anterior regions (Figure 7D and H), and substantially increasing 3F11 signal intensity (Figure 7D). Thus, for the three mAbs we tested, reduction had the most beneficial effect. However, since antigen-antibody interactions can respond more or less favorably to individual treatments [113], we suggest that all three treatments be included in efforts to optimize antibodies with low signal, high background, or poor penetration.

### mAbs label planarian tissues in unbleached histological sections

Histological sections provide superior spatial resolution of cellular events in some contexts [17,43,116-118], and reduce the potential impact of issues such as antibody penetration in thick specimens. Furthermore, sections also allow easy visualization of internal tissues without requiring bleaching, an appealing option since peroxide treatment can be detrimental (Figures 4 and 5).

**Figure 8:**
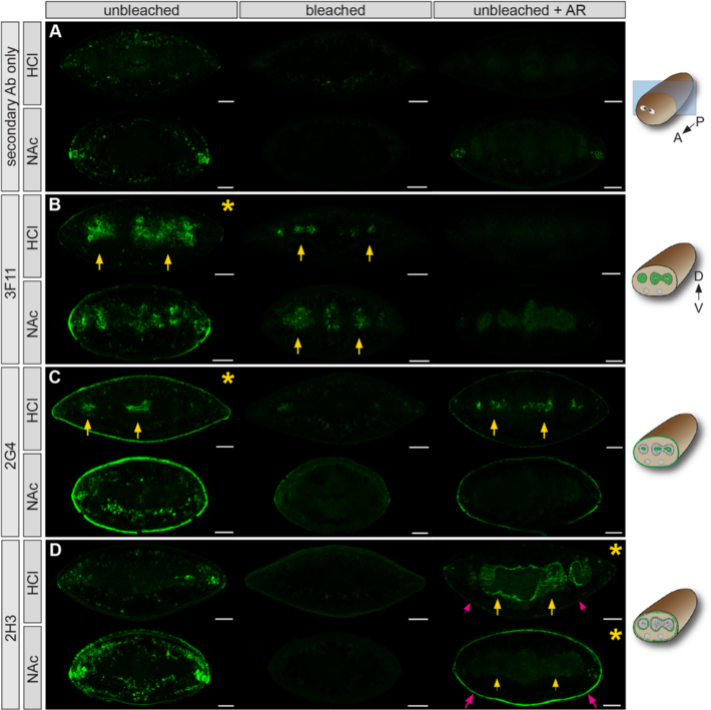
**mAb labeling of histological sections.** Cryosections (20 μm) from formaldehyde-fixed planarians processed as indicated. **(A)** Sections labeled with secondary antibody only. **(B)** mAb 3F11 labels the intestine (arrows). **(C)** 2G4 labels the apical regions of intestinal cells (arrows) and epidermis. **(D)** 2H3 labels enteric (yellow arrows) and body wall (magenta arrows) muscles. Schematics illustrating orientation of cross sections and tissues labeled (green) are to located the right of each set of panels. All planarians were treated with HCl or NAc, and fixed in formaldehyde/Triton X-100 (20 min). Bleaching in 6% H_2_O_2_/PBS (12 hr) (middle column) was conducted prior to cryosectioning. Antigen retrieval was conducted after sectioning. Dorsal is to the top in all images. Conditions yielding the highest signal-to-noise for each mAb are indicated with an asterisk. Scale bars: 100 μm.

We assessed labeling by our mAbs on cryosections from HCl- and NAc-treated, formaldehyde-fixed planarians, and compared sections generated from both bleached and unbleached animals (Figure 8 and Additional file 6; Table 3). Additionally, we assessed whether antigen retrieval enabled detection of epitopes on unbleached sections, since AR is widely used to unmask antigens in formaldehyde-fixed, paraffin-embedded tissue [115].

**Table 3:** **Effects of sample processing on immunolabeling of histological sections**

**mAb**	**Fixation protocol**									
	**HCl**	**NAc**	**HCl**	**NAc**	**HCl**	**NAc**	**HCl**	**NAc**	**HCl**	**NAc**
	**Formaldehyde**									
	**-**		**Bleach**		**AR**		**Reduction**		**Prot K**	
2G4, int.	++	+	+	-	++	-	+	+	-	-
2G4, ep.	++	++	-	-	+	+	-	-	+	+
3F11	++	+	+	++	-	-	+	+	+	-
2C11	+	+	++	+	++	+	+	-	+	-
2D2	+	-	-	-	-	-	+	-	-	-
2H3	-	-	-	-	++	+	-	-	+	-
3H3	++	++	++	++	++	++	+	+	+	+
**mAb**	**Methacarn**									
2G4, int.	+	+	-	n.d.	++	++	+	-	+	-
2G4, ep.	+	+	-	n.d.	-	-	++	+	++	+
3F11	-	-	-	n.d.	-	-	n.d.	n.d.	n.d.	n.d.
2C11	+	++	++	n.d.	-	++	+	+	+	++
2D2	-	-	-	n.d.	-	-	n.d.	n.d.	n.d.	n.d.
2H3	-	-	-	n.d.	+	+	-	-	-	-
3H3	++	++	++	n.d.	++	++	++	++	+	+

Qualitative assessment of signal:noise for tissues labeled by selected mAbs: 2G4, intestine (“int.”) and epidermis (“ep.”); 3F11, intestine; 2H3, enteric muscle; 2D2, CNS neuropil; 2C11, peripharyngeal secretory cells and projections; 3H3, epidermis. “-“ indicates negligible or non-uniform labeling, poor morphology, and/or high background. “+“ indicates moderate specificity, uniform labeling, or strong signal accompanied by elevated non-specific labeling. “++” indicates robust signal with minimal noise. “n.d.”, not done.

In sections labeled with secondary antibody alone (Figure 8A), background labeling was minimal, but higher in NAc-treated sections; both bleaching and AR reduced this background significantly. mAb 3F11, the antibody that was most sensitive to bleaching (Figure 5G-J), labeled the intestine robustly in unbleached sections from both HCl- and NAc-treated animals (Figure 8B). Background labeling of non-intestinal tissue was higher in NAc-treated sections, although much of this labeling was likely non-specific, since it also occurred in sections labeled with secondary antibody alone (Figure 8A). In sections from bleached animals, non-specific labeling was reduced, but intestinal signal in 3F11-labeled sections also decreased, especially in HCl-treated samples (Figure 8B). AR was as effective as bleaching at reducing non-specific labeling (Figure 8A), but also abolished 3F11 intestinal labeling (Figure 8B), regardless of mucus removal method. We also co-labeled sections from HCl-treated, NAc-fixed, unbleached animals with the lectin *Lens culinaris* agglutinin (LCA), which labels intestinal goblet cells (Additional file 6: A-C) [48]. Overlap with 3F11 labeling was minimal, suggesting 3F11 recognizes intestinal phagocytes with a high degree of specificity.

Demonstrating the variable sensitivity of antigens to processing, labeling by 2G4 (Figure 8C and Additional file 6: D) was affected differently by the same treatments. First, 2G4 labeled the lumenal region of the intestine much more intensely in HCl-treated samples than NAc-treated samples. Second, bleaching virtually eliminated 2G4 labeling. Third, antigen retrieval preserved intestinal labeling in HCl-treated samples, while greatly reducing non-specific mesenchymal background as well as specific epidermal signal.

One antibody, 2H3, labeled both subepidermal body wall muscles (Figure 1O) and visceral muscles surrounding intestinal branches (Figure 3E), in patterns strikingly similar to previous studies using both phalloidin and other muscle-specific antibodies [17,58,59]. Further illustrating the utility of testing multiple parameters together, 2H3 only labeled sections after AR treatment (Figure 8D and Additional file 6: E). Interestingly, in HCl-treated sections, visceral muscle labeling was high, while external muscle labeling was low. The opposite effect was observed in NAc-treated samples, in which body wall muscles were labeled more intensely than visceral muscles. Thus, the 2H3 epitope may be sensitive to HCl treatment, and is degraded more quickly in external than internal tissues during mucus removal.

Other antibodies displayed a similar range of treatment optima. For example, 2D2 labeled neuronal projections only in unbleached, HCl-treated, non-AR sections (Additional file 7: A). 2C11 labeled peripharyngeal secretory cells and their projections after all six treatment combinations, but signal was most specific and highest in bleached, HCl-treated sections (Additional file 7: B). 3H3 was the least sensitive to processing, intensely labeling the epidermis in all samples (Additional file 7: C).

We tested other post-fixation treatments (reduction and proteinase K digestion), and also tested antibodies on sections from methacarn-fixed planarians (Table 3). As expected, mAbs responded variably to these treatments. Although in no case was labeling more specific than in formaldehyde-fixed samples, AR treatment did enable muscle detection by 2H3 in methacarn-fixed sections (Table 3), suggesting that some epitopes benefit from heat treatment, even in the absence of cross-linking fixation.

To summarize, optimal mAb labeling of histological sections usually requires a specific combination of sample preparation parameters, as in whole animals. Although we initially selected for mAbs that would label peroxide-bleached animals in our primary screen (Figure 1), we nonetheless found that on sections, labeling by a number of antibodies was completely abolished by bleaching. One possible explanation for this result is that additional processing steps (cryoembedding, freezing, air drying, thawing, and rehydration), together with the detrimental effects of peroxide bleaching, combine to degrade or alter epitopes in a way that prevents their detection. Finally, for some antibodies, AR is an effective method for reducing background and enabling antigen detection on sections from unbleached planarians. AR is therefore a viable alternative to bleaching, since epitopes that are irreversibly damaged by peroxide treatment may be preserved in heat-treated histological sections.

### A systematic approach to optimization of sample processing in planarians

We have shown that the specificity of antibodies raised against planarian tissue can be affected by every step of sample processing. Our observations are consistent with an extensive body of immunohistochemical research demonstrating that the molecular complexity of antigens makes their sensitivity to chemical treatments unique and unpredictable [86,88,89,119,120]. We screened our initial hybridoma library on HCl-treated, formaldehyde-fixed, PBS-bleached planarians. While we successfully identified antibodies that labeled under these conditions, some mAbs performed better or worse when planarians were processed differently. Thus, although the axiom “you get what you screen for” holds true, screening itself does not automatically identify optimal processing conditions. Furthermore, our data imply that when screening antibodies raised against specific molecules (e.g., fusion proteins), preparing samples several ways may be advisable. For example, while we favor formaldehyde fixation because of its faithful preservation of protein localization and cellular morphology [103,104], the effects of HCl or NAc on specific epitopes likely need to be tested empirically. Alternatively, screening could be conducted to identify antibodies that label robustly in specific applications, for example, on samples that have first undergone *in situ* hybridization.

**Figure 9:**
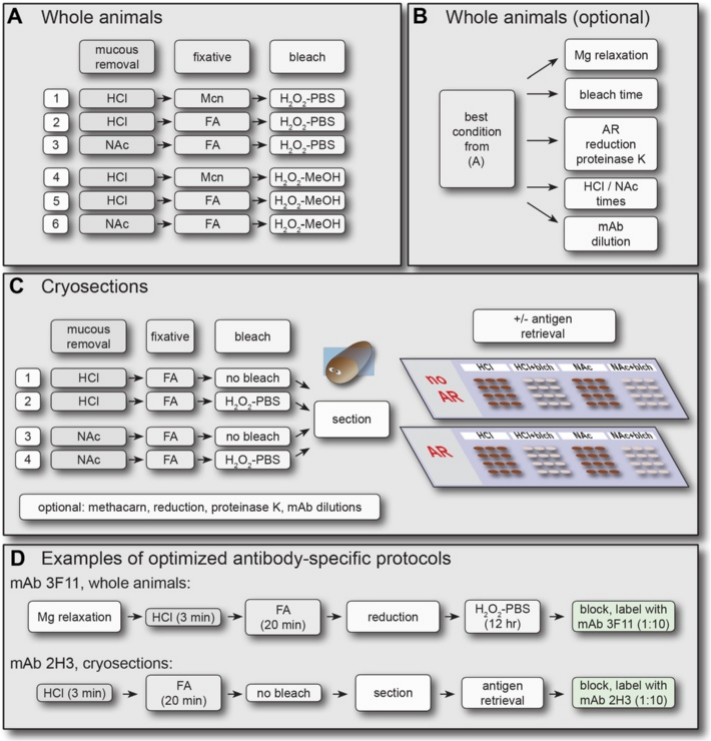
**A combinatorial method for optimization of antibody labeling in planarians. (A)** Schematic of first round optimization steps for whole animals. **(B)** Schematic of potential further optimization steps. **(C)** Optimization steps on cryosections. **(D)** Examples of optimized protocols for antibodies raised in this study.

Although development of a universal protocol suitable for every antibody is likely impossible, testing a limited number of methods together can efficiently identify conditions that preserve antigenicity and achieve excellent signal-to-noise for many antibodies (Figure 9). For whole planarians, we have developed a two stage optimization workflow (Figure 9A and B). In the first stage (Figure 9A), mucolytic agents, fixatives, and bleaching diluents are tested in combination for a total of six initial conditions. Although we did not extensively test methacarn together with milder NAc treatment, such a combination could be included, increasing the total number of initial conditions to eight. In an optional second stage (Figure 9B), other parameters such as magnesium-induced relaxation, bleaching time, antigen retrieval, and antibody dilution can be tested to further refine a protocol for a particular antibody, as we have done for mAbs 3F11, 2D2, and 2C11. Alternative approaches are also possible. For example, reduction and proteinase K treatment could be tested during the first round, increasing the number of initial combinations to 18.

Because peroxide bleaching affects some antigens detrimentally, and because some antigens may require unmasking, we also recommend testing mAbs on cryosections from bleached and unbleached planarians in combination with antigen retrieval (Figure 9C). We routinely adhere sections from planarians prepared four different ways to the same slide. Using two slides, eight combinations including antigen retrieval can be analyzed simultaneously in one experiment. Again, parameters can be easily substituted; for example, bleached samples could be omitted in favor of samples fixed in methacarn, or reduction could be tested instead of antigen retrieval.

**Figure 10:**
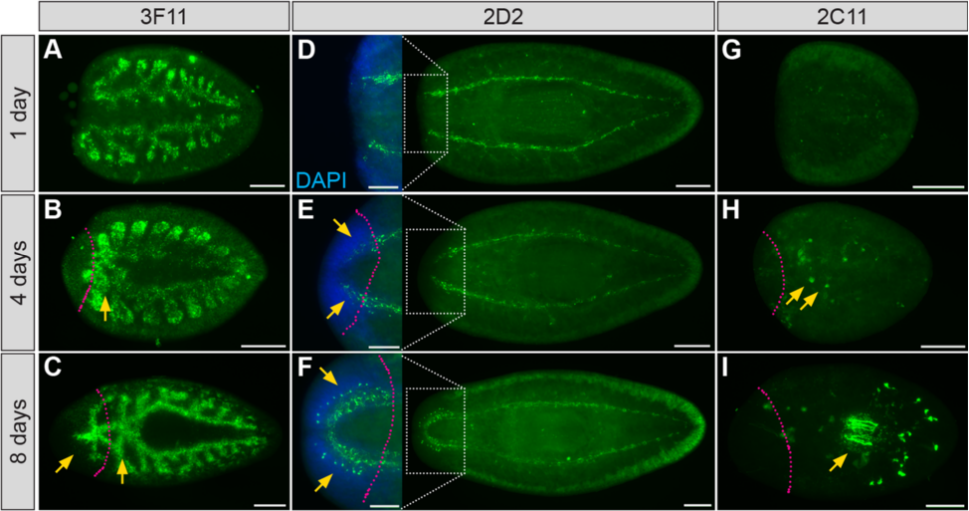
**mAbs label regenerating intestine**, **secretory cells**, **and central nervous system in specimens processed using optimized protocols. (A**-**C)** 3F11 labels the intestine in tail fragments regenerating a new head, including anterior regions that remodel (arrow, **B**) and elongate (arrows, **C**) at early and intermediate stages of intestinal regeneration, respectively. **(D**-**F)** After head amputation, 2D2 labels the regenerating brain, including new neuronal projections (arrows, **E**), and cell bodies (arrows, **F**). **(G**-**I)** 2C11 labels the regenerating peripharyngeal secretory system in tail fragments, including the first appearance of cell bodies 4 days after amputation (arrows, **H**), and projections within the pharynx 8 days after amputation (arrow, **I**). Days after amputation are indicated at left. Anterior is to the left in all panels. Dotted magenta lines indicate approximate boundary between new tissue (left) and old tissue (right). All samples were processed using the optimized protocol for each mAb described in Additional file 5. Scale bars: 100 μm (insets in **D**-**F**); 200 μm (all others).

By approaching sample preparation systematically, we have substantially improved processing protocols for several of the monoclonal antibodies we generated (examples in Figure 9D; Tables 2 and 3). These protocols are suitable for use on uninjured animals as well as regenerates, and enable visualization of intestinal remodeling and growth (Figure 10A-C), re-establishment of the central nervous system after head amputation (Figure 10D-F), and the *de novo* development of secretory cells and their projections into the pharynx (Figure 10G-I). We have assembled step-by-step protocols detailing each stage of sample preparation, including examples of initial optimization steps for whole animals, as well as summary protocols for several individual antibodies (Additional file 2).

### Future directions

Although we evaluated many of the more commonly used fixatives and sample preparation conditions, our analysis was not exhaustive. Alternative fixatives, mucus removal treatments, bleaching agents, and tissue permeabilization methods remain to be tested or might emerge in the future. For example, a combined relaxing agent and fixative has been used previously to prepare planarians for paraffin embedding, sectioning, and immunolabeling [107,121]. The modular nature of our optimization workflow allows straightforward substitution or addition of such treatments. Additionally, although we conducted tests of the duration of bleaching and mucus removal, we have not rigorously explored treatment time as a parameter. In particular, fixation and permeabilization may need to be extended for larger planarians, as noted for *in situ* hybridization protocols [34]. Similarly, initial trials suggest that small animals and tissue fragments (<2 mm) may be more sensitive to hydrogen peroxide treatment, requiring bleaching for 10 hr or less, at least for some antibodies.

## Conclusions

We raised a panel of mAbs against planarian intestinal cells, and identified optimal sample preparation conditions for several antibodies. Serendipitously, our screen also yielded markers for non-intestinal cell types, reagents that may benefit studies of regeneration of the nervous system, pharynx, and epidermis. Our results reinforce the feasibility of producing tissue-specific markers using whole planarian cells as an immunogen. Furthermore, our approach to testing multiple parameters together during sample processing should accelerate future efforts to develop planarian-specific antibodies, and to extend investigation of regenerative mechanisms to post-transcriptional aspects of gene expression including protein localization and modification. Finally, our observations emphasize the long-appreciated sensitivity of antibody specificity to fixation and other treatments, and the utility of systematically testing multiple approaches when addressing organism-specific idiosyncracies such as mucus secretions or tissue permeability. Our experiences and approaches may facilitate efforts to develop immunological resources in other emerging model organisms.

## Methods

### Planarian care and maintenance

Asexual *Schmidtea mediterranea* (clonal line ClW4) were maintained in 0.5 g/L Instant Ocean salts as described [122]. Phagocytes were collected from large (>9 mm) planarians. Animals 5-7 mm in length were utilized for cryosections. For all other experiments, small planarians (3-5 mm) were used. Animals were starved for 5-10 days prior to experiments.

### Phagocyte collection and fixation

Eighty to 100 planarians were fed a mixture of Feridex (AMAG Pharmaceuticals), liver homogenate, ultra-low melting point agarose, and food coloring (Durkee) as described [23]. 36-48 hours later, animals were dissociated in calcium- and magnesium-free medium with BSA (“CMF”) [58,123] and 0.6 U/ml Dispase (Invitrogen) [23], and filtered sequentially through 160 μm, 53 μm, and 30 μm nylon meshes in Swinnex filters (Millipore). After each filtration, cells were pelleted at 150 × *g* for 5 min, then resuspended in fresh CMF.

After the final spin, cells were resuspended in 2 ml degassed CMF + 0.5 mM EDTA (“CMF-E”), and applied to a Miltenyi LS Column mounted on a VarioMACS cell separator. Column equilibration and purification were conducted according to the manufacturer’s protocol, except that degassed CMF-E was used as the buffer for all steps, and phagocyte elution was conducted with three 3 ml CMF-E elutions by gravity flow, which was gentler and yielded more intact cells than plunger flushing of the column.

Phagocytes were pelleted at 300 × *g* for 5 min, then immediately fixed for 10 minutes in either 4% formaldehyde/1X PBS (RT) or 67% methanol/33% PBS (-20°C). Cells were rinsed three times in 1X PBS, then incubated overnight (O/N) in 1X PBS/0.1% Triton X-100 at 4°C. After three rinses in 1X PBS, cells were stored at 4°C. Equal numbers of FA-fixed and MeOH-fixed cells were pooled and supplied in PBS to the University of Illinois Immunological Resources Facility for immunization. Each purification routinely yielded 1-3 × 10^5^ phagocytes; approximately 50 collections total were conducted.

### Immunization, serum tests, hybridoma generation, and screening

All animals were obtained and cared for in strict accordance with the policies and guidelines of the Division of Animal Resources (DAR) and the Institutional Animal Care and Use Committee (IACUC) of the University of Illinois. The DAR was responsible for all animal veterinary care. Immunization, fusion, hybridoma generation, and cloning were conducted by the University of Illinois Immunological Resources Facility (IACUC protocol 07082).

BALB/c female mice were injected three times (every three weeks) with 5 × 10^5^ to 1 × 10^6^ fixed phagocytes per injection, with a final, fourth boost of 9 × 10^5^ cells. Antigen emulsion was made by mixing cell suspension with an equal volume of adjuvant. Titermax adjuvant and incomplete Freund’s adjuvant were used for primary immunization and subsequent immunizations, respectively.

Preimmune sera were drawn prior to immunization, while test bleeds were drawn after the third immunization. Both were tested at 1:100, 1:200, 1:500, and 1:1000 in blocking solution on fixed, bleached planarians using previously described methods [17]. Briefly, planarians were killed in ice-cold 2% HCl for 5 min, then fixed for 6 hr in either 4% formaldehyde (FA)/1X PBS, Carnoy’s fixative (6:3:1 ethanol:chloroform:glacial acetic acid), or methacarn (6:3:1 methanol:chloroform:glacial acetic acid) at 4°C. FA-fixed animals were bleached O/N (16 hr) in 6% H_2_O_2_/1X PBS; Carnoy’s- and methacarn-fixed animals were bleached in 6% H_2_O_2_/methanol. Animals were incubated for 4 hr at RT in BSA/fish gelatin blocking solution [17], and O/N at 4°C in test sera and secondary antibody (goat anti-mouse 568, Molecular Probes, used at 1:1000), which were diluted in blocking solution. 6-8 washes in PBSTx (0.3% Triton X-100 in 1X PBS) were conducted over >6 hr at RT after primary and secondary incubations.

The best immune responders to intestinal cells were sacrificed and their lymphocytes fused with Sp2/0 myeloma cells to generate hybridoma cell lines following standard protocols [80]. For the primary screen, planarians were fixed in formaldehyde and immunolabeling was performed as for serum testing, except that incubations were carried out in 96 well plates, and supernatants were used undiluted. Fusions secreting specific antibodies were selected, subcloned, and re-screened to generate final hybridoma cell lines. Modified standard HAT medium containing 10% FBS was used to culture hybridoma cells in a 7% CO_2_ incubator.

Antibody-producing lines have been deposited in the Developmental Studies Hybridoma Bank, Iowa City, IA. Although re-optimization of dilution is recommended, we have validated DSHB-produced supernatants from multiple lines (3G9, 3F11, 2G4, 2H3, 2D2, and 2C11), and find that they perform identically to those produced at Illinois. We also note that although IgM antibodies can be less stable than IgG mAbs [65,99], in our hands supernatants from IgM-producing hybridomas were stable for a minimum of 12 months when stored at 4°C, regardless of production site.

### Isotyping

Antibodies were isotyped using the IsoStrip Mouse Monoclonal Antibody Isotyping Kit (Roche), according to the manufacturer’s instructions.

### Magnesium-induced relaxation

Planarians were placed in a glass vial (Research Products International) in 1 ml planarian salts. Two ml 1M MgCl_2_ (RT) were quickly added to the vial (0.66 M final concentration), and animals were gently swirled for 15-30 sec. After animals had uncurled and relaxed, 10 ml 1X PBS were quickly added. After animals had settled at the bottom of the tube, the salts/MgCl_2_/PBS solution was removed completely, allowing animals to flatten against the sides and bottom of the vial. Hydrochloric acid or N-Acetyl-cysteine solution (below) was then added immediately.

### Mucus removal (HCl)

Animals were placed in glass vials and chilled on ice in planarian salts for 1 minute (animals relaxed in MgCl_2_ were not pre-chilled). Planarian salts were removed and replaced with ice-cold 2% HCl (i.e., 36-38% HCl diluted 1:18) in water (v/v); vials were shaken moderately by hand for 1 min, chilled on ice for 1 min, shaken again for 1 min, and then HCl was removed. Prior to formaldehyde fixation, animals were rinsed quickly in 1X PBS, which was then removed completely before fixative was added. Alternatively, methacarn was added directly after HCl treatment.

### Mucus removal (NAc)

For N-Acetyl-L-cysteine treatment [44], animals were placed in glass vials, planarian salts were removed, and 7.5% NAc (Sigma)/1X PBS (w/v) was added. Animals were gently rocked for 7 min (RT), then NAc was removed completely and replaced with fixative.

### Methacarn fixation and bleaching

Ice-cold methacarn (6:3:1 methanol:chloroform:acetic acid) was added directly to animals after mucus removal. Fixation was conducted at 4°C with gentle rocking. Planarians were fixed for 20 minutes. Fixative was then removed and replaced with -20°C methanol. Samples were rocked for 10-15 minutes at 4°C, and rinsed twice more in methanol (RT). Animals were then bleached in 6% H_2_O_2_ in methanol (in a foil-lined container 6-10 cm below a fluorescent light at RT, 14-20 hr unless noted otherwise), rinsed 3X in methanol, then stored at -20°C or rehydrated immediately for experiments. Animals were rehydrated by incubation in 1:1 methanol:PBSTx (v/v) (5 min), followed by three washes in PBSTx (5 min each). We note that in preliminary studies, we also tested antibodies on animals fixed in the related Carnoy's fixative as well as methanol alone. However, we found that results were comparable or worse than methacarn, and did not test these fixatives further.

### Formaldehyde fixation and bleaching

For optimization of whole-animal immunolabeling, planarians were fixed in 4% formaldehyde (EMD Biosciences)/PBSTx (1X PBS plus 0.3% Triton X-100) for 20 min. For cryosections, planarians were fixed in 4% FA (Ted Pella 18505)/PBSTx for 20 min. Fixations were conducted with gentle rocking at RT. After fixation, animals were rinsed 3X in PBSTx.

For whole-animal optimization, planarians were then dehydrated by incubation in 1:1 methanol:PBS (v/v) (5 min) followed by two incubations in methanol at RT, permeabilized for >1 hr in methanol at -20°C, then bleached overnight (14-20 hr, unless noted otherwise) in 6% H_2_O_2_ in methanol at RT. After bleaching, animals were rinsed several times in methanol, then stored at -20°C or rehydrated and used immediately. Bleaching in PBS was conducted similarly, except that animals were rinsed twice more in 1X PBS, bleached in 6% H_2_O_2_ in 1X PBS, rinsed 3X in 1X PBS, and stored (<5 days) at 4°C or used immediately.

### Reduction (whole planarians)

After formaldehyde fixation but prior to bleaching, planarians were incubated in reduction solution (50 mM DTT, 1% NP-40, and 0.5% SDS, in 1X PBS) for 10 min at 37°C [34], with occasional gentle agitation. Animals were then washed 3X in PBSTx (5 min each).

### Proteinase K treatment (whole planarians)

Bleached animals were incubated in 10 μg/ml proteinase K (Invitrogen) in PBSTx + 0.1% SDS [34] for 10 min with gentle rocking (RT). Planarians were rinsed in PBSTx three times, post-fixed in 4% FA/PBSTx for 10 min, then rinsed three more times in PBSTx.

### Antigen retrieval (whole planarians)

Bleached planarians were equilibrated in 10 mM sodium citrate (pH 6.0) (5 min). Fresh sodium citrate was added, then animals were incubated in a heat block at 95-100°C (10 min). Planarians were allowed to cool to RT, then washed 2X in PBSTx (5 min each).

### Immunofluorescence (whole planarians)

Planarians were equilibrated in PBSTx (5 min), then blocked O/N (16-20 hr) in BSA/fish gelatin blocking solution (0.6% IgG-free BSA (Jackson Immuno) and 0.45% fish gelatin (Sigma) in PBSTx) at RT. Supernatants were diluted 1:2 in blocking solution (i.e., one volume supernatant and one volume blocking buffer) in Figures 2 and 3, and Additional files 3, 4, and 5. In Figures 6 and 7, mAb 3F11 was diluted 1:2. In all other figures, supernatants were diluted 1:10 or 1:100 (2C11 only). Planarians were incubated O/N at 4°C. After 6-8 PBSTx washes over at least 6 hr, planarians were re-blocked for 1-2 hr at RT, then incubated with goat anti-mouse IgG + IgM HRP (Jackson Immuno) at 1:250 and DAPI (1 μg/ml) O/N at 4°C. For direct detection, goat anti-mouse IgG + IgM Dylight-488 (Jackson Immuno) was used at 1:500. After incubation in secondary antibody, animals were washed 6-8X in PBSTx over at least 6 hr, then twice in PBSTw (0.01% Tween-20 in 1X PBS) (5 min each). For TSA, planarians were incubated with FITC-Tyramide [34] at 1:1500 plus 0.005% H_2_O_2_ in PBSTw for 10-15 min at RT, then washed 3X in PBSTx (10 min each). Planarians were washed overnight in PBSTx, rinsed again in PBSTx, then mounted in Vectashield.

### Cryosectioning

Fixed, bleached planarians (rehydrated if necessary) were cryoprotected in 15% sucrose (w/v)/PBS (>10 min at RT) followed by 30% sucrose/PBS (O/N at 4°C). Animals were stored for up to two weeks at 4°C in 30% sucrose prior to cryosectioning.

Samples were transferred to Tissue Freezing Medium (TBS) in silicone “Pelco” EM molds (Ted Pella), frozen on dry ice, and stored for up to one week at -80°C. Blocks were cryosectioned at 20 μm on a Microm HM550 cryostat, and sections adhered to Superfrost Plus slides (Fisher) coated with gelatin (0.5%) and chromium potassium sulfate (0.05%). Slides were air-dried at RT for 1-3 hr, then stored for up to two weeks at -80°C prior to immunolabeling.

### Rehydration (cryosections)

Slides were warmed to room temperature (5-10 min), then incubated in three changes of 1X PBS (>5 min each, RT) in Coplin jars to rehydrate and remove tissue freezing medium.

### Proteinase K treatment (cryosections)

After rehydration, slides were equilibrated in PBSTx (5 min). 50 μl of a solution of 10 μg/ml proteinase K (Invitrogen) in PBSTx + 0.1% SDS was added to each slide, then slides were coverslipped and incubated for 10 min (RT). Slides were then quickly dipped in PBSTx in a Coplin jar to remove coverslips, then slides were briefly incubated (15-30 sec) in two rinses of PBSTx. Sections were post-fixed in 50 μl 4% FA/PBSTx, then washed 3X in PBSTx in Coplin jars (5 min each).

### Reduction (cryosections)

After rehydration, slides were equilibrated in PBSTx (5 min). 50 μl of reduction solution was added to each slide, then slides were coverslipped and incubated for 10 min at 37°C. Slides were dipped in PBSTx in a Coplin jar to remove coverslips, then washed 3X in PBSTx (5 min each).

### Antigen retrieval (cryosections)

After rehydration, slides were equilibrated in 10 mM sodium citrate (pH 6.0) (5 min, RT), then transferred to fresh sodium citrate in microwave-safe plastic Coplin jars. Slides in sodium citrate were heated to boiling in a microwave, then brought back to boiling every minute for 10 minutes total; care was taken to minimize superheating. Slides were then allowed to cool gradually to room temperature.

### Immunofluorescence (cryosections)

Slides were equilibrated in PBSTx (5 min), blocked for 30 min in low-volume plastic slide jars (Ted Pella 21096), then incubated with mAb supernatants under glass coverslips for 2 hr in a humidified staining chamber. All supernatants were diluted 1:10 (one part supernatant, nine parts block) in blocking solution except 2C11, which was diluted 1:50. Coverslips were removed in PBSTx, rinsed in PBSTx (<30 sec) to remove excess antibody, and washed three times in PBSTx (10 min each). Slides were then incubated for 2-3 hr with HRP-conjugated goat anti-mouse IgG + IgM (Jackson) at 1:250 and DAPI at 1 μg/ml. Coverslips were removed and slides were rinsed and washed as above in PBSTx. Following two washes in PBSTw (5 min each), TSA was performed on slides with FITC-tyramide (1:1500) in PBSTw with 10 min development. After TSA, slides were washed at least three times in PBSTx (10 min each), then mounted in Vectashield. Rhodamine-LCA (Vector Laboratories) was incubated with secondary antibody at 1.25 μg/μl. All antibody incubations and TSA were conducted on-slide, in 50 μl volumes under 22 × 50 mm glass coverslips; all washes were carried out in glass Coplin jars. All steps were conducted at RT.

### Imaging and image processing

Samples were imaged on a Zeiss SteREO Lumar.V12 running AxioVision (v4.6.3 and later), a Nikon Eclipse TE200 with a MicroFIRE camera (Optronics) and Picture Frame v2.3, a Zeiss Axio Observer.A1 with a Retiga 4000R camera (QImaging) and QCapture Suite PLUS (v3.1.3.10), or a Zeiss LSM710 laser scanning confocal running Zen. Images were processed using ImageJ 1.46r [124] and Adobe Photoshop CS4. Where appropriate, exposure times were kept constant and adjustments to brightness and contrast were applied identically to allow comparison of immunolabeling.

## Additional files

Additional file 1:
**Detailed protocol for isolation of planarian intestinal phagocytes.**
Additional file 2:
**Detailed protocols for fixation and immunofluorescent labeling of planarians.**
Additional file 3:
**Comparison of direct to indirect detection and effects of blocking duration on immunofluorescent labeling.** (A-C) mAb detection using DyLight 488-conjugated secondary antibody. (D-F) mAb detection using HRP-conjugated secondary and FITC-tyramide (tyramide signal amplification). (G-I) mAb labeling after blocking for 4 hr prior to immunolabeling. (J-L) mAb labeling after blocking overnight (16-20 hr) prior to immunolabeling. For 3F11, animals were relaxed in magnesium, treated with 2% HCl, fixed in formaldehyde, and bleached in PBS. For 2C11 and 2D2, animals were processed identically except mucus was removed with 7.5% NAc and animals were bleached in methanol. For TSA vs. fluorophore-conjugated secondary comparison, animals were blocked overnight. For blocking comparison, TSA was used for detection. mAbs are indicated at the top of the figure. Scale bars: 100 μm (B’, E’, H’, K’); 500 μm (all other panels). Additional file 4:
**Optimization of additional mAbs.** (A) mAb 1H8 labeling is enriched in central nervous system nuclei, especially in NAc-treated samples. (B) mAb 1E12 labels nuclei. Signal is most robust after methacarn fixation; in combination with magnesium relaxation, epidermal signal is dramatically reduced, revealing intestinal nuclear labeling. Sample treatment parameters are indicated at left. In all panels, anterior is to the left. Yellow asterisks indicate conditions that yielded the most specific signal with minimal background labeling. All samples were bleached in methanol (16-20h). Scale bars: 100 μm (A); 500 μm (B). Additional file 5:
**Excessive bleaching adversely affects mAb labeling.** (A-D) NAc-treated planarians labeled with mAb 3F11 after bleaching for the times indicated. (E-H) Planarians labeled with mAb 3G9 after bleaching for the times indicated. Planarians were relaxed in magnesium chloride, treated with 7.5% NAc (3F11) or 2% HCl (3G9), fixed in formaldehyde/Triton X-100 (3F11) or methacarn (3G9), and bleached in 6% H_2_O_2_/PBS (3F11) or 6% H_2_O_2_/methanol (3G9). Scale bars: 500 μm. Additional file 6:
**mAb labeling of cryosections.** (A) Cryosection labeled with mAb 3F11, Rhodamine-LCA, and DAPI. (B-C) High magnification of the region boxed in (A) shows the minimal overlap in labeling between 3F11 (phagocytes) and LCA (goblet cells). Asterisks indicate examples of 3F11-negative goblet cells. (D) 2G4 labeling of the apical region of the intestine (arrows) and ventral epidermis. (E) 2H3 labeling of muscle fibers around the intestine (arrows). 2H3 also weakly labels ventral body wall muscles under these conditions. 3F11- and 2G4-labeled samples were HCl-treated, FA-fixed, without AR. 2H3-labeled samples were HCl-treated, FA-fixed, and AR-treated. (A, D, E) Confocal projections of 20 μm thick cryosections. (B, C) 2 μm optical sections of the region boxed in (A). Scale bars: 100 μm (A, D, E); 20 μm (B, C). Additional file 7:
**mAb labeling of non-intestinal tissues on histological sections.** Cryosections (20 μm) from formaldehyde-fixed planarians prepared as indicated and labeled with the antibodies shown. (A) Sections through the planarian brain labeled with mAb 2D2. Arrows indicate neuronal projections within cephalic ganglia (cg, dotted lines, and inset) in sections from HCl-treated, unbleached animals. (B) mAb 2C11 labels peripharyngeal secretory cells and their projections (arrows) in sections through the pharyngeal region. (C) mAb 3H3 labels epidermis. For all images, planarians were treated with HCl or NAc, and fixed in formaldehyde/Triton X-100 (20 min). Bleaching in 6% H_2_O_2_/PBS (12 hr) (middle column) was conducted prior to cryosectioning. Antigen retrieval was conducted after sectioning, on-slide, prior to immunolabeling. Dorsal is to the top in all images. Scale bars: 100 μm.

## Abbreviations

mAb: monoclonal antibody
TSA: tyramide signal amplification
IgM: immunoglobulin M
IgG: immunoglobulin G
IHC: immunohistochemistry
HCl: hydrochloric acid
NAc: N-Acetyl-L-cysteine
FA: formaldehyde
Mcn: methacarn
AR: antigen retrieval
