# Supplementary material for: Generation of cell type-specific monoclonal antibodies for the planarian and optimization of sample processing for immunolabeling
Source: BMC Dev Biol. 2014 Dec 21;14:45. doi: 10.1186/s12861-014-0045-6 (PMC4299570; doi:10.1186/s12861-014-0045-6)
Supplement: Additional file 1: — Detailed protocol for isolation of planarian intestinal phagocytes. [file 12861_2014_45_MOESM1_ESM.docx]

**Additional File 1: Detailed protocol for isolation of planarian intestinal phagocytes**

David J. Forsthoefel, Forrest A. Waters, and Phillip A. Newmark

Contents:

Feeding magnetic beads...................2

Dissociation.......................................3

Magnetic separation..........................4

Working Solutions.............................5

Reagents and Equipment..................5

References........................................6

**Feeding Magnetic Beads**

1. One day before feeding, transfer 50-100 large asexual planarians (>8 mm in length) to fresh salts with gentamicin at 50 μg/ml. Animals should be starved for 5-7 days prior to feeding.

2. Aliquot the following “soft-serve” mix in a microcentrifuge tube:

20 μl basic microbeads (Miltenyi)

176 μl liver homogenate (2:1 liver paste:ultra-pure water)

4 μl food coloring (Durkee)

3. Gently vortex to mix, then spin down (<2,000 x *g* for 5-10 sec).

4. Pipet into container of planarians. All animals will eat in 30-60 min.

5. Remove excess food and rinse animals thoroughly. Transfer to fresh salts with gentamicin in a clean container, and remove non-eating animals.

**Planarian Dissociation**

1. 24 to 48 hr after animals were fed magnetic beads, rinse in fresh planarian salts and transfer to Petri dishes.

2. Rinse animals briefly in 10 ml CMF [[1-3](#_ENREF_1)] and remove.

3. Cut each animal into 2-3 pieces; add 10 ml CMF and gently rock for 5 minutes.

4. Transfer fragments to 2-3 microcentrifuge tubes, remove CMF and replace with 500 μl CMF+Dispase.

5. Homogenize briefly with a small Kontes pestle, then pool homogenates in 15 ml polypropylene tubes in CMF+Dispase (10 ml total volume).

6. Gently rock, triturating every 5-10 minutes^1^. Dissociation is usually complete within 20-30 min at RT.

7. Filter through 160 μm nylon mesh^2^, centrifuge filtrate at 200 x *g* for 5 min, and discard supernatant.

8. Gently resuspend cell pellet in 5-10 ml CMF; repeat step 7 with 53 μm and 30 μm meshes.

9. Resuspend final pellet in 2 ml of degassed CMF-E^3^.

Notes:

^1^ Avoid bubbles and treat the cells as gently as possible throughout the dissociation.

^2^ Mesh is mounted in Swinnex-25 filter units (Millipore).

^3^ CMF-E is degassed by pulling a vacuum in a clean side-arm flask for 5-10 minutes. This step prevents bubble accumulation in the column during magnetic separation.

**Magnetic separation of intestinal phagocytes**

1. Mount an LS column on a Miltenyi VarioMACS separator.

2. Equilibrate LS column by applying 3 ml degassed CMF-E and letting buffer run through. Discard effluent and change collection tube.

3. Resuspend filtered cell pellet (from up to 100 large asexual planarians) in 2 ml degassed CMF-E.

4. Apply cell suspension to equilibrated LS column.

5. Collect non-intestinal (non-bead-containing) cells that flow through. Wash column with 3 x 3 ml CMF-E, adding buffer each time column reservoir is empty, yielding a total of ~11 ml eluted cells.

6. Remove LS column from separator and mount on a ring stand/clamp assembly over a new collection tube.

7. Collect intestinal (bead-containing) cells with 3 x 3 ml CMF-E, allowing cells to elute from the column by gravity flow^1^.

8. Spin at 300 x *g* for 5 minutes to pellet cells for downstream applications.

Note:

^1^ Do not use the plunger supplied with the column; this method of elution causes excessive disintegration of a significant portion of intestinal phagocytes.

**Working Solutions**

CMF: 15 mM HEPES pH 7.4; 400 mg/L NaH_2_PO_4_; 800 mg/L NaCl; 1200 mg/L KCl; NaHCO_3_; 240 mg/L D-glucose; 1% BSA

CMF+Dispase: CMF with Dispase II/Neutral Protease (Gibco/Invitrogen) at 0.6 U/ml final activity. Small aliquots of 100X stock solution can be stored at 4°C for <1 week.

CMF-E: CMF plus 0.5 mM EDTA

**Reagents and Equipment**

| **Description** | **Manufacturer** | **Catalog Number/ID** |
| --- | --- | --- |
| Gentamicin Sulfate | Gemini Bio-Products | 400-108 |
| Basic Microbeads | Miltenyi Biotec | 130-048-001 |
| BSA | Sigma-Aldrich | A3912 |
| Dispase II | Gibco/Invitrogen | 17105-041 |
| Swinnex-25 filter holders | Millipore | SX0002500 |
| Nylon Mesh, 160 μm | Elko/Sefar | 03-160/53 |
| Nylon Mesh, 53 μm | Elko/Sefar | 03-53/30 |
| Nylon Mesh, 30 μm | Elko/Sefar | 03-30/18 |
| LS Columns | Miltenyi Biotec | 130-042-401 |
| "VarioMACS" magnet | Miltenyi Biotec | 130-090-282 |

**References for Additional File 1**

1. Baguñà J: **Estudios citotaxonómicos, ecológicos e histofisiología de la regulación morfogenética durante el crecimiento y la regeneración en la raza, asexuada de la planaria *Dugesia mediterranea*.** *PhD thesis, Universitat de Barcelona, Spain* 1973.

2. Bueno D, Baguñà J, Romero R: **Cell-, tissue-, and position-specific monoclonal antibodies against the planarian *Dugesia (Girardia) tigrina***. *Histochem Cell Biol* 1997, **107**(2):139-149.

3. Reddien PW, Oviedo NJ, Jennings JR, Jenkin JC, Sánchez Alvarado A: **SMEDWI-2 is a PIWI-like protein that regulates planarian stem cells**. *Science* 2005, **310**(5752):1327-1330.
