# Supplementary material for: Generation of cell type-specific monoclonal antibodies for the planarian and optimization of sample processing for immunolabeling
Source: BMC Dev Biol. 2014 Dec 21;14:45. doi: 10.1186/s12861-014-0045-6 (PMC4299570; doi:10.1186/s12861-014-0045-6)
Supplement: Additional file 2: — Detailed protocols for fixation and immunofluorescent labeling of planarians. [file 12861_2014_45_MOESM2_ESM.docx]

**Additional File 2: Detailed protocols for fixation and immunofluorescent labeling of planarians**

David J. Forsthoefel, Forrest A. Waters, and Phillip A. Newmark

Contents:

Magnesium relaxation...........................................................2

N-Acetyl cysteine mucus removal.........................................3

HCl mucus removal...............................................................3

Methacarn fixation.................................................................4

Formaldehyde fixation...........................................................4

Reduction..............................................................................5

Hydrogen peroxide bleaching...............................................6

Proteinase K treatment..........................................................7

Antigen retrieval.....................................................................7

Immunolabeling.....................................................................8

Tyramide Signal Amplification (TSA).....................................9

Cryosectioning.....................................................................10

Gelatin/CPS slide coating....................................................12

Rehydration (cryosections)..................................................13

Antigen retrieval (cryosections)...........................................13

Reduction (cryosections).....................................................13

Proteinase K treatment (cryosections).................................14

Immunolabeling (cryosections)............................................15

Sample whole animal optimization.......................................16

Sample whole animal labeling (mAb 3F11)..........................17

Sample cryosection protocol (mAb 3F11)............................18

Optimal protocols for selected mAbs.(whole animals).........19

Optimal protocols for selected mAbs.(cryosections)............20

Working Solutions................................................................21

Reagents/Equipment............................................................22

References...........................................................................23

**Magnesium relaxation**

1. Place 5-10 animals in a small glass vial, or 20-30 in a large vial.

2. Remove all but 0.5ml/1ml planarian salts (small/large vial).

3. While animals are moving, add 1ml/2ml 1M MgCl_2_ (final conc. = 0.67M).

4. Swirl gently for 15-30 sec maximum. Animals will first contract, then relax and uncurl.

5. Once animals have uncurled^1^, fill remainder of vial with 1X PBS and swirl. Animals will sink and collect in the center of the bottom.

6. Quickly^2^ but carefully remove *all* MgCl_2_/PBS, so that most animals flatten on the bottom/sides of the vial (use a transfer pipet followed by a P100 pipet for the last few drops). Proceed *immediately* to HCl or NAc mucus removal.

Notes:

^1^ Usually only 80-90% of animals will properly uncurl/flatten, so start with more than are needed for the experiment.

^2^ The entire Mg step must be completed within 90-120 sec or animals will begin to lyse due to (presumably) osmotic shock.

**Mucus removal with N-Acetyl Cysteine** (Adapted from [[1](#_ENREF_1)])

(skip step 1 if animals have been relaxed in magnesium)

1. Place 5-10 animals in a small glass vial, or 20-30 in a large vial. Remove salts.

2. Add 5 ml/10 ml (small/large vial) 7.5% NAc/PBS. Incubate with gentle rocking for 7 min.

3. Remove NAc.

4. Add fixative.

**Mucus removal with HCl** (Adapted from [[2](#_ENREF_2)])

(skip steps 1-2 if animals have been relaxed in magnesium)

1. Place 5-10 animals in a small glass vial, or 20-30 in a large vial.

2. Incubate animals on ice 30 sec – 2 min, until movement slows considerably. Quickly remove salts.

3. Add 5 ml/10 ml (small/large vial) ice-cold 2% HCl (diluted into ultra-pure water).

4. Shake 1 min, incubate on ice 1 min, shake for final minute (3 min total).

5. Quickly remove all HCl. If fixing in formaldehyde, quickly rinse animals in 1X PBS^1^ and remove.

6. Add fixative.

Note:

^1^ This step prevents interaction between HCl and formaldehyde to form the carcinogen bis-chloromethyl ether (BCME).

**Methacarn fixation**

1. Add 5ml/10ml (small/large vial) ice-cold methacarn.

2. Shake moderately by hand for 1 min, or until animals sink.

3. Rock gently for 20 min at 4°C.

4. Remove methacarn, add -20°C methanol, rock 15-30 min at 4°C.

5. Remove methanol, rinse animals 1-2 times in methanol, then proceed with bleaching or storage at -20°C.

**Formaldehyde fixation**

1. Add 5 ml/10 ml (small/large vial) 4% formaldehyde solution.

2. Rock gently at RT for 20 min.

3. Remove fixative, then rinse 3 times in PBSTx, 5 min each^1^.

4. Proceed with storage^1^ or bleaching.

Notes:

^1^ Animals can be stored for up to a week in PBSTx at 4°C, or dehydrated into methanol and stored indefinitely at -20°C in methanol. Storage effects should be tested for individual antibodies. See Note 1 in “Hydrogen peroxide bleaching” (page 5) for details on dehydration.

**Reduction** (Adapted from [[3](#_ENREF_3)])

1. Rinse animals in PBSTx^1^.

2. Add freshly-made reduction solution, incubate at 37°C for 10 min with gentle inversion every 2-3 minutes.

3. Wash 3 times with PBSTx, 5 min per wash.

4. Proceed with bleaching.

Note:

^1^ Reduction is conducted after fixation, prior to bleaching. Animals can be transferred to standard 1.5 ml polypropylene microcentriguge tubes for this step.

**Hydrogen peroxide bleaching**

1. Add 6% H_2_O_2_/methanol or 6% H_2_O_2_/PBS to animals, invert several times to mix.^1^

2. Bleach in a tinfoil-lined container, 10-15 cm under a fluorescent lamp, for 8-24 hr^2^.

3. Rinse animals 3 times in methanol or PBS, 5 min each.

4. Store animals at -20°C (in methanol) or at 4°C (in PBS), or proceed with rehydration (for animals in methanol) and immunolabeling.

Note:

^1^ Dehydration/rehydration: If necessary, rehydrate animals by washing in 1:1 methanol:PBSTx (5 min), followed by two washes in PBSTx, 5 min each. Dehydrate animals by washing in methanol:PBSTx (5 min), followed by two washes in methanol, 5 min each. For example, after methacarn fixation, animals should first be rehydrated into PBS before bleaching in 6% H_2_O_2_/PBS. Conversely, after FA fixation, dehydrate animals prior to bleaching in 6% H_2_O_2_/methanol.

^2^ Bleaching time should be optimized for animal size, fixation conditions, and individual antibodies.

**Proteinase K treatment** (Adapted from [[3](#_ENREF_3)])

1. If necessary, rehydrate animals by washing in 1:1 methanol:PBSTx (5 min), followed by two washes in PBSTx, 5 min each.

2. Add proteinase K solution, incubate with gentle rocking at RT for 10 min^1^.

3. Remove proteinase K, and rinse animals 2-3 times in PBSTx.

4. Immediately post-fix for 10 min in 4% formaldehyde solution.

5. Rinse animals 3 times in PBSTx, 5 min each.

Note:

^1^ Optimized for intact asexuals, 3-5 mm. Proteinase K concentration and/or treatment time may need to be adjusted for larger or smaller animals, or for specific antibodies.

**Antigen retrieval**

1. If necessary, rehydrate animals^1^ (see Step 1, above, for proteinase K treatment.)

2. Equilibrate animals 2 times in AR solution at RT, 5 min each.

3. Incubate animals in AR solution in a heat block at 100°C for 10 min.

4. Allow animals to cool gradually to RT.

5. Rinse animals 2 times in PBSTx, 5 min each.

Note:

^1^ AR can be performed before or after bleaching. We have not tested AR extensively on whole planarians. Furthermore, although AR exposes antigens on cryosections, we have not yet identified antigens that benefit from this treatment in whole (bleached) planarians.

**Immunolabeling**

1. Rehydrate animals if needed (1:1 methanol:PBSTx for 5 min, then 2 washes in PBSTx, 5 min each).

2. Incubate 4-16 hr^1^ in blocking solution, at RT.

3. Dilute mAb (1:1-1:100, as appropriate)^2^ in blocking solution^3^.

4. Incubate animals in mAb solution for 12-16 hr with gentle rocking at 4°C.

5. Remove mAb solution, wash animals 6-8 times in PBSTx over >6 hr at RT.

6. Reblock 30-60 min in blocking solution.

7. Mix secondary antibody solution: HRP-conjugated goat anti-mouse IgG+IgM (H+L) 1:250 (Jackson), plus DAPI at 1 μg/ml in blocking buffer.

8. Incubate animals in secondary antibody solution for 12-16 hr with gentle rocking at 4°C.

9. Wash animals 6-8 times in PBSTx over >6 hr at RT.

10. Proceed with tyramide signal amplification.

Notes:

^1^ We routinely block “overnight” (12-16 hr), which moderately improves labeling with intestinal antibodies. Non-intestinal antibodies can be used after blocking for 4 hr, but longer is not detrimental.

^2^ See Supplemental Table 1. Optimal dilution should be determined for each batch of supernatant.

^3^ 75-100 μl solution is sufficient for 3-4 small asexuals in one well of a 96 well plate.

**Tyramide Signal Amplification**

(Adapted from [[3-5](#_ENREF_3)])

1. Wash twice in PBSTw, 5 min each.

2. Add tyramide solution to animals (100 μl minimum in 96 well plates).

3. Incubate in dark for 10 minutes^1^.

4. Remove tyramide solution.

5. Wash 3 times in PBSTx, 10 min each, then overnight at 4°C.

6. Rinse in PBSTx, and mount in Vectashield or other medium.

Note:

^1^ Tyramide-only preincubation and/or longer development times may improve labeling in animals over 5 mm in length.

**Cryosectioning**

1. Cryoprotect. In a polypropylene microcentrifuge tube, incubate appropriately fixed and processed animals in 15% sucrose solution for 10 min at RT. Replace with 30% sucrose solution, incubate for 10 min at RT or overnight at 4°C^1^.

2. Mount. Fill the well of a silicone embedding mold (e.g., “Pelco 106” from Ted Pella) with TBS Tissue Freezing Medium ("mounting medium")^2^.

3. Transfer 1-4 planarians to the surface of the mounting medium (use a pipet tip cut with a razor blade). Remove as much excess 30% sucrose as possible.

4. Orient animals appropriately with a tungsten needle or other probe (see diagram below). Remove excess mounting medium so that surface is flat or slightly convex.

5. Freeze entire mold on a flat block of dry ice immediately^3^.

6. Cryosection^4^. Apply mounting medium to metal chuck. Remove individual blocks from the silicone mold, orient appropriately, and freeze to the chuck (see diagram below). Cut sections (typically 20 μm) and apply to gelatin/CPS coated SuperFrost Plus slides. Air-dry slides for 1 – 3 hr (i.e., as long as it takes to section).

6. Store slides in a plastic slide box (Ted Pella), sealed in a ziplock bag, at -80°C^5^.

Notes:

^1^ For best results, cryoprotect planarians immediately after fixation or bleaching. Animals can be stored at 4°C for several weeks, although some antigens might be more or less sensitive to storage.

^2^ We have found that “TFM” curls less than the more widely used OCT, particularly for small blocks. Animals can be equilibrated in freezing medium prior to mounting, but this step has little effect on morphology or immunolabeling.

^3^ Frozen blocks can be stored at -80°C indefinitely for many antibodies.

^4^ Equipment and procedures vary among institutions. Accordingly, we have provided only a general outline. For small blocks, we have found that sectioning at -25°C reduces curling and yields more consistent sections.

^5^ We have successfully labeled sections after three months of storage at -80°C.

**Cryosectioning (continued)**


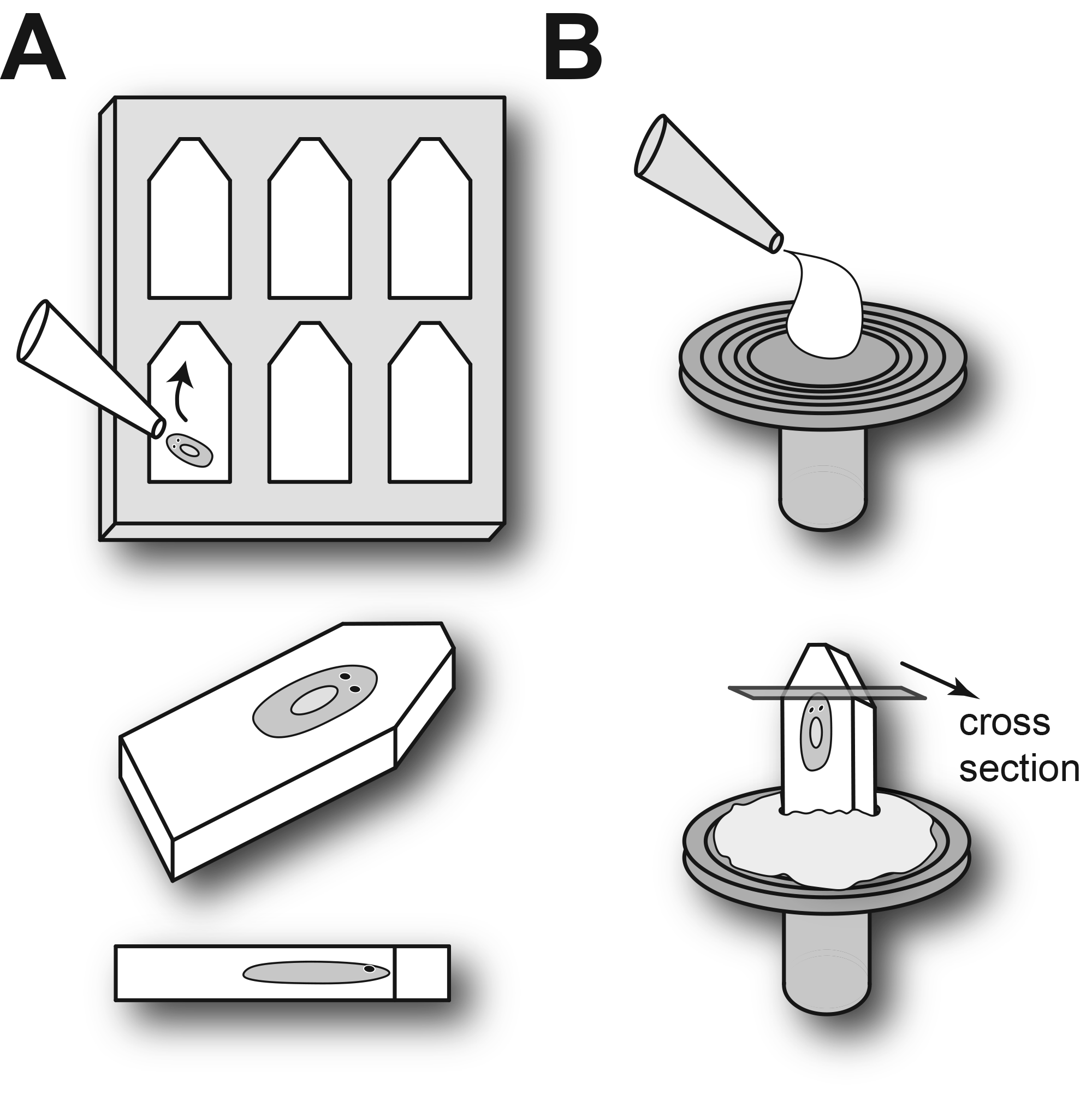


**Mounting cryosections.** (**A**) Fixed planarians are transferred to a well of a silicone mold filled with mounting medium, then oriented so that the anterior tip of the animal aligns with the tapered end of the well/block. (**B**) Mounting medium is added, and the block is frozen to the chuck so that the animal is positioned perpendicularly to the cutting plane (to generate cross sections).

**Gelatin/CPS slide coating**

1. Heat 500 ml of distilled water in a microwave to 40°C.

2. Add 2.5 gm of gelatin; stir to dissolve.

3. Add 250 mg of chromium (III) potassium sulfate (“CPS”); stir to dissolve^1^.

4. Mount several boxes of SuperFrost Plus slides in staining racks (e.g., Ted Pella 21078).

5. Pour gelatin/CPS solution into staining box (e.g. Ted Pella 21078-1).

6. Dip slides for 5-10 sec and dab away excess on paper towels. Air-dry slides overnight^2^.

Notes:

^1^ Remove excess foam from the solution using a glass rod and Kim wipes prior to coating slides.

^2^ Performing this step in a hood will minimize dust contamination on slides.

**Cryosection rehydration**

1. Remove slides from -80°C, allow to equilibrate to room temperature for 10 min.

2. Rehydrate sections (and dissolve mounting medium) in 1X PBS in glass staining jars: 3 washes, 5-10 min each.

**Antigen retrieval (slides)**

1. Rehydrate sections, then equilibrate in AR solution (5 min).

2. Transfer slides to AR solution in plastic, microwave-safe Coplin jars.

3. Microwave. Heat solution just until boiling begins, being very careful not to superheat or allow AR solution to boil over. (Heating on lower power can also minimize superheating.) Then heat every minute for 5-10 sec to bring back to just boiling. Repeat for 10 min.

4. Allow slides to cool to RT gradually (30-45 min).

5. Wash slides 2 times in PBSTx, 5 min each.

**Reduction (slides)**

1. Rehydrate sections, then equilibrate in PBSTx (5 min).

2. Remove slide from staining jar, and quickly dab away excess PBSTx from bottom and sides of slide with a Kimwipe.

3. Add 50 μl reduction solution, and cover sections with 22x50 mm glass coverslips, using fine forceps.

4. Incubate at 37°C for 10 min.

5. Dip slides in PBSTx in staining jars to remove coverslips.

6. Wash 3 times in PBSTx, 5 min each.

**Proteinase K treatment (slides)**

1. Rehydrate sections, then equilibrate in PBSTx (5 min).

2. Remove slide from staining jar, and quickly dab away excess PBSTx from bottom and sides of slide with a Kimwipe.

3. Add 50 μl Proteinase K solution, and cover sections with 22x50 mm glass coverslips, using fine forceps.

4. Incubate at RT for 5 min.

5. Dip slides in PBSTx in staining jars to remove coverslips.

6. Rinse 2 times in PBSTx, 15-30 sec each.

7. Post-fix immediately with 50 μl 4% formaldehyde solution, 5 min.

8. Remove coverslips, wash 3 times in PBSTx, 5 min each.

**Immunolabeling (cryosections)**

1. Equilibrate slides in PBSTx (5 min) in a glass staining jar.

2. Incubate slides in blocking solution for 30 min in a plastic staining jar.

3. Dilute mAb into blocking solution, 50 μl per slide.

4. Remove slides from staining jar, and dab away excess blocking solution with a Kimwipe. Add mAb solution (50 μl), and cover sections with a 22x50 mm cover glass using fine forceps.

5. Incubate in a humidified staining chamber^1^ for 2 hr at RT.

6. Gently remove coverslips by dipping in PBSTx. Rinse slides in fresh PBSTx, 15-30 sec, to remove excess antibody.

7. Wash slides 3 times in PBSTx, 10 min each.

8. Dab away excess PBSTx, add secondary antibody solution (HRP-conjugated goat anti-mouse IgG+IgM, 1:250, plus DAPI at 1 μg/ml), 50 μl per slide, under a coverslip.

9. Incubate in a humidified staining chamber for 2 hr at RT.

10. Remove coverslips, rinse slides, and wash 3 times in PBSTx, 10 min each, as above.

11. Wash slides 2 times in PBSTw, 5 min each.

12. Dab away excess PBSTx, add 50 μl tyramide solution and coverslip. Incubate 10 min at RT.

13. Remove coverslips, rinse slides, and wash 3 times in PBSTx, 10 min each.

14. Cover sections with Vectashield (~22 μl) and 22x50 mm cover glass.

Note:

^1^ For example, a plastic “Tuff Tainer” with a moistened paper towel.

**Sample optimization protocol (whole animals)**

The following is a sample protocol that evaluates 6 combinations of mucus removal, fixation, and bleaching diluent for two different mAb supernatant concentrations. Eight small (3-5 mm) asexual planarians are fixed per combination, then divided into two wells of a 96 well plate for immunofluorescent labeling. 48 animals total are used.

1. Process 6 sets of samples^1^, 8 animals per vial:

| **Abbreviation** | **Mucus Removal** | **Fixation** | **Bleach** |
| --- | --- | --- | --- |
| HM-P | 2% HCl (3 min) | Methacarn (20 min) | 6% H_2_O_2_/PBS (12 hr) |
| HF-P | 2% HCl (3 min) | 4% Formaldehyede (20 min) | 6% H_2_O_2_/PBS (12 hr) |
| NF-P | 7.5% NAc (7 min) | 4% Formaldehyede (20 min) | 6% H_2_O_2_/PBS (12 hr) |
| HM-M | 2% HCl (3 min) | Methacarn (20 min) | 6% H_2_O_2_/MeOH (12 hr) |
| HF-M | 2% HCl (3 min) | 4% Formaldehyede (20 min) | 6% H_2_O_2_/MeOH (12 hr) |
| NF-M | 7.5% NAc (7 min) | 4% Formaldehyede (20 min) | 6% H_2_O_2_/MeOH (12 hr) |

2. Wash, rehydrate (MeOH-bleached samples), and aliquot each set of animals to two wells of a 96 well plate (4 animals per well), 12 wells total:


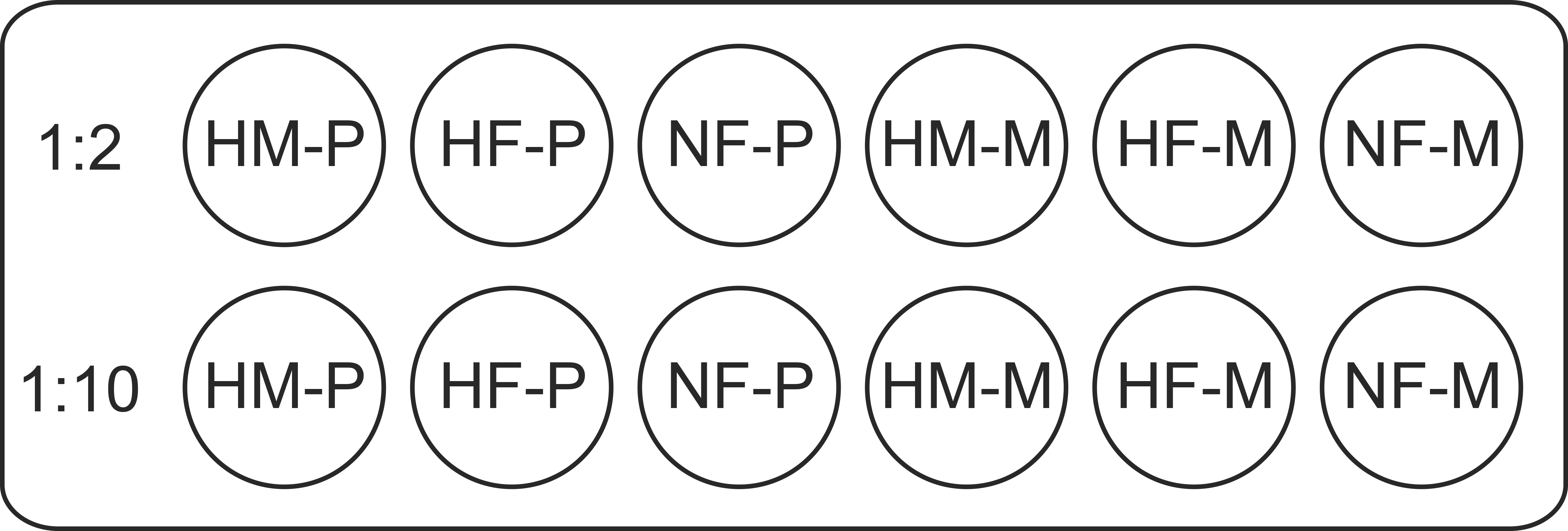


4. Block 4-16 hr.

5. Prepare mAb solutions:

(a) 1:2, 6 wells, 100 μl per well = 300 μl supernatant + 300 μl blocking solution

(b) 1:10, 6 wells, 100 μl per well = 60 μl supernatant + 560 μl blocking solution

6. Remove block, add mAb. Incubate overnight, 4°C.

7. Remove mAb, rinse wells with PBSTx, then wash 6 times in PBSTx, 1 hr each.

8. Re-block 1 hr.

9. Incubate in goat anti-mouse HRP, 1:250, plus DAPI at 1 μg/ml (100 μl/well).

10. Rinse all wells, then wash 6 times in PBSTx, 1 hr each.

11. Wash 2 times in PBSTw.

12. TSA, 10 min.

13. PBSTx washes.

14. Mount in Vectashield.

Note:

^1^ For simplicity, abbreviated protocols are shown that do not include washes, etc.

**Sample Protocol, mAb 3F11 (whole animals)**

Processing:

1. Relax 3-5 mm asexual planarians in MgCl_2_.

2. Remove mucus in 2% HCl (3 min), then rinse quickly in PBS.

3. Fix in 4% formaldehyde/PBSTx, 20 min.

4. Wash 3 times in PBSTx, 5 min each.

5. Reduce animals, 10 min at 37°C.

6. Wash 3 times in PBSTx, then twice in PBS, 5 min per wash.

7. Bleach for 12 hr in 6% H_2_O_2_.^1^

8. Wash 3 times in PBSTx.

Immunolabeling:

1. Block 16 hr.

2. Incubate in mAb 3F11 (1:10 in blocking solution), overnight at 4°C.

3. Rinse in PBSTx, then wash 6 times, 1 hr each, in PBSTx.

4. Re-block 1 hr.

5. Incubate in goat anti-mouse HRP 1:250 plus DAPI 1 μg/ml, overnight at 4°C.

6. Rinse in PBSTx, then wash 6 times, 1 hr each, in PBSTx.

7. Wash 2 times in PBSTw, 5 min each.

8. TSA, 10 min.

9. Wash 3 times in PBSTx, 10 min each, then overnight.

10. Final rinse in PBSTx, then mount in Vectashield.

Note:

^1^ Optionally, store unbleached animals overnight at 4°C prior to bleaching.

**Sample Protocol, mAb 2H3 (cryosections)**

Processing:

1. Remove mucus in 2% HCl (3 min).

2. Fix in 4% formaldehyde/PBSTx, 20 min.^1^

3. Wash 3 times in PBSTx, 5 min each.

4. Incubate in 15% sucrose/1X PBS.

5. Incubate in 30% sucrose/1X PBS.

Cryosectioning:

1. Mount animals in tissue freezing medium.

2. Cryosection at 20 μm.

3. Store slides at -80°C.

Rehydration and AR:

1. Warm slides to RT, rehydrate in 1X PBS (3 x 5-10 min each).

2. Equilibrate in AR solution, 5 min, then microwave slides in AR solution, 10 min.

3. Cool gradually to RT, equilibrate in PBSTx.

Immunolabeling:

1. Block 30 min.

2. Incubate in mAb 2H3 (1:10 in blocking solution), 2 hr at RT.

3. PBSTx washes – rinses to remove coverslip and excess mAb, then 3 x 10 min.

4. Incubate in goat anti-mouse HRP 1:250 plus DAPI 1 μg/ml, 2 hr at RT.

5. PBSTx washes – rinses to remove coverslip and excess mAb, then 3 x 10 min.

6. PBSTw washes – 2 x 5 min.

7. TSA, 10 min.

8. PBSTx washes – rinses to remove coverslip and excess tyramide, then 3 x 10 min.

9. Mount in Vectashield.

Note:

^1^ Use freshly diluted 16% formaldehyde from Ted Pella.

**Optimal protocols for selected mAbs (whole animals)**

| **Clone** | **Dilution^a^** | **Relaxation/**  **Mucus Removal** | **Fixation** | **Bleach** |
| --- | --- | --- | --- | --- |
| 2G4 | 1:10 | Mg; 2% HCl (3 min) | methacarn (20 min) | 6% H_2_O_2_/  MeOH (12-16 hr) |
| 3F11 | 1:10 | Mg; 2% HCl (3 min) | 4% FA (20 min); reduction | 6% H_2_O_2_/  PBS (12 hr) |
| 3G9 | 1:4 | Mg; 2% HCl (3 min) | methacarn (20 min) | 6% H_2_O_2_/  MeOH (12 hr)** |
| 4D2 | 1:2* | Mg; 2% HCl (3 min) | methacarn (20 min) | 6% H_2_O_2_/  MeOH (12-16 hr)** |
| 1E12^b^ | 1:2* | Mg; 2% HCl (3 min) | methacarn (20 min) | 6% H_2_O_2_/  MeOH (12-16 hr)** |
| 1H8 | 1:2* | Mg; 7.5% NAc (7 min) | 4% FA (20 min) | 6% H_2_O_2_/  MeOH (12-16 hr)** |
| 2C11 | 1:100 | Mg; 7.5% NAc (7 min) | 4% FA (20 min) | 6% H_2_O_2_/  MeOH (12-16 hr) |
| 2D2 | 1:10 | Mg; 7.5% NAc (7 min) | 4% FA (20 min) | 6% H_2_O_2_/  MeOH (12-16 hr) |
| 2H3 | 1:10 | -/+ Mg;  7.5% NAc (7 min) | 4% FA (20 min) | 6% H_2_O_2_/  MeOH (12-16 hr)** |
| 3G7 | 1:2* | 2% HCl (3 min) | 4% FA (20 min) | 6% H_2_O_2_/  MeOH (12-16 hr)** |
| 3H3 | 1:10 | +/- Mg; 2% HCl (3 min) | methacarn (20 min) | 6% H_2_O_2_/  MeOH (12-16 hr)** |

^a^ Highest dilution tested, but may vary for unique batches of supernatant.

^b^ Optimal protocol to visualize intestinal nuclei.

* Dilution was not optimized.

** PBS bleaching was not tested.

**Optimal protocols for selected mAbs (cryosections)**

| **Clone** | **Dilution^a^** | **Mucus Removal** | **Fixation** | **Bleach^b^** | **AR** |
| --- | --- | --- | --- | --- | --- |
| 2G4 | 1:10 | 2% HCl (3 min) | 4% FA (20 min) | no | optional |
| 3F11 | 1:10 | 2% HCl (3 min)  -or-  7.5% NAc (7 min) | 4% FA (20 min) | no (with HCl)  -or-  yes (with NAc) | no |
| 2C11 | 1:50 | 2% HCl (3 min) | 4% FA (20 min) | yes | no |
| 2D2 | 1:10 | 2% HCl (3 min) | 4% FA (20 min) | no | no |
| 2H3 | 1:10 | 2% HCl (3 min)* | 4% FA (20 min) | no | yes |
| 3H3 | 1:10 | 2% HCl (3 min)  -or-  7.5% NAc (7 min) | 4% FA (20 min)  -or-  methacarn (20 min) | optional | optional |

^a^ Highest dilution tested, but may vary for unique batches of supernatant.

^b^ Bleaching (if specified) was performed in 6% H_2_O_2_/PBS (12 hr).

^c^ Best protocol for 2H3 labeling of visceral muscle.

**Working Solutions**

7.5% NAc/PBS: Dissolve 7.5 g N-Acetyl-L-cysteine (Sigma) into 10 ml 1X PBS.

2% HCl: Dilute 0.556 ml 36% HCl into 9.5 ml ultra-pure water (1:18 dilution).

Methacarn: For 10 ml, mix 6 ml methanol, 3 ml chloroform, and 1 ml glacial acetic acid. Methacarn solution can be stored for 1-2 weeks at 4°C.

4% formaldehyde solution: For whole animal fixation, dilute 1 ml 36.5% methanol-stabilized formaldehyde solution (EMD/Millipore) into 8 ml PBSTx. For animals that will be cryosectioned, mix 10 ml 16% formaldehyde (Ted Pella), 4 ml 10X PBS, and 1.2 ml 10% Triton X-100. Store at 4°C for <2 weeks.

PBSTx: 1X PBS plus 0.3% Triton X-100

Reduction solution: 50 mM DTT, 1% NP-40, 0.5% SDS, 1X PBS

6% H_2_O_2_/methanol: Dilute 2 ml 30% H_2_O_2_ into 8 ml methanol.

6% H_2_O_2_/PBS: Dilute 2 ml 30% H_2_O_2_ into 8 ml 1X PBS.

Proteinase K solution: 10 μg/ml proteinase K (Invitrogen) in PBSTx plus 0.1% SDS

AR solution: 10 mM sodium citrate, pH 6.0 (can dilute from filter sterilized 100 mM stock)

Blocking solution: 0.6% (w/v) IgG-free BSA (Jackson), 0.45% (v/v) fish gelatin (Sigma), 1X PBS, 0.3% Triton X-100

4.5% fish gelatin stock solution: Warm 45% fish gelatin to 50°C, dilute 1:10 in ultra-pure water, store at 4°C, re-warm prior to further dilution.

PBSTw: 1X PBS plus 0.01% Tween-20

0.5% H_2_O_2_: 30% H_2_O_2_ stock diluted 1:60 into PBSTw.

Tyramide solution: FITC-Tyramide diluted 1:1500 plus 0.5% H_2_O_2_ diluted 1:100 (final concentration 0.005%) into PBSTw (e.g., 1 μl FITC-Tyramide, 15 μl 0.5% 0.5% H_2_O_2_, 1.5 ml PBSTw). FITC-Tyramide was synthesized as in [[5](#_ENREF_5)].

15% sucrose solution: 15% sucrose (w/v) in 1X PBS.

30% sucrose solution: 30% sucrose (w/v) in 1X PBS.

**Reagents and Equipment**

| **Description** | **Manufacturer** | **Catalog Number/ID** |
| --- | --- | --- |
| ***Processing:*** | | |
| Glass Vials (small) | RPI | 125505 |
| Glass Vials (large) | RPI | 121002 |
| HCl | JT Baker/VWR | 9535-03 |
| N-Acetyl-L-cysteine | Sigma-Aldrich | A7250 |
| 36.5% formaldehyde | EMD/Millipore | FX0410-5 |
| 16% formaldehyde | Sigma | 18505 |
| 30% H_2_O_2_ | Sigma | H1009 |
| Proteinase K solution | Invitrogen | 25530-049 |
| Sodium Citrate | Fisher | S279 |
| ***Labeling*:** | | |
| IgG-free BSA | Jackson ImmunoResearch | 001-000-162 |
| 45% fish gelatin | Sigma-Aldrich | G7765 |
| Peroxidase-conjugated goat anti-mouse IgG+IgM (H+L) | Jackson ImmunoResearch | 115-035-044 |
| DAPI | Sigma-Aldrich | D9542 |
| Vectashield | Vector Laboratories | H-1000 |
| ***Cryosectioning*:** | | |
| Pelco 106 embedding molds | Ted Pella | 106 |
| TBS Tissue Freezing Medium | Ted Pella | 27209 |
| SuperFrost PLUS slides | Fisher | 12-550-15 |
| Handy Mini Slide Box | Ted Pella | 2108 |
| Gelatin | Sigma-Aldrich | G2500 |
| Chromium (III) Potassium Sulfate | Sigma-Aldrich | C337 |
| ***Labeling cryosections:*** | | |
| Glass staining jars | Ted Pella | 432-1 |
| Polypropylene Coplin jars | Ted Pella | 21029 |
| 22x50 mm No. 1 glass coverslips | Fisher/Gold Seal | 3317 |
| Slide Staining Jar (low volume) | Ted Pella | 21096 |
| “Tuff Tainer” Slide Staining Box | US Plastic | 53669 |

**References for Additional File 2**

1. Gurley KA, Rink JC, Sánchez Alvarado A: **β-catenin defines head versus tail identity during planarian regeneration and homeostasis**. *Science* 2008, **319**(5861):323-327.

2. Umesono Y, Watanabe K, Agata K: **A planarian *orthopedia* homolog is specifically expressed in the branch region of both the mature and regenerating brain**. *Dev Growth Differ* 1997, **39**(6):723-727.

3. Pearson BJ, Eisenhoffer GT, Gurley KA, Rink JC, Miller DE, Sánchez Alvarado A: **Formaldehyde-based whole-mount in situ hybridization method for planarians**. *Dev Dyn* 2009, **238**(2):443-450.

4. Wang Y, Zayas RM, Guo T, Newmark PA: **nanos function is essential for development and regeneration of planarian germ cells**. *Proc Natl Acad Sci U S A* 2007, **104**(14):5901-5906.

5. King RS, Newmark PA: **In situ hybridization protocol for enhanced detection of gene expression in the planarian Schmidtea mediterranea**. *BMC Dev Biol* 2013, **13**:8.
