# Supplementary figures and images for: Generation of cell type-specific monoclonal antibodies for the planarian and optimization of sample processing for immunolabeling
Source: BMC Dev Biol. 2014 Dec 21;14:45. doi: 10.1186/s12861-014-0045-6 (PMC4299570; doi:10.1186/s12861-014-0045-6)

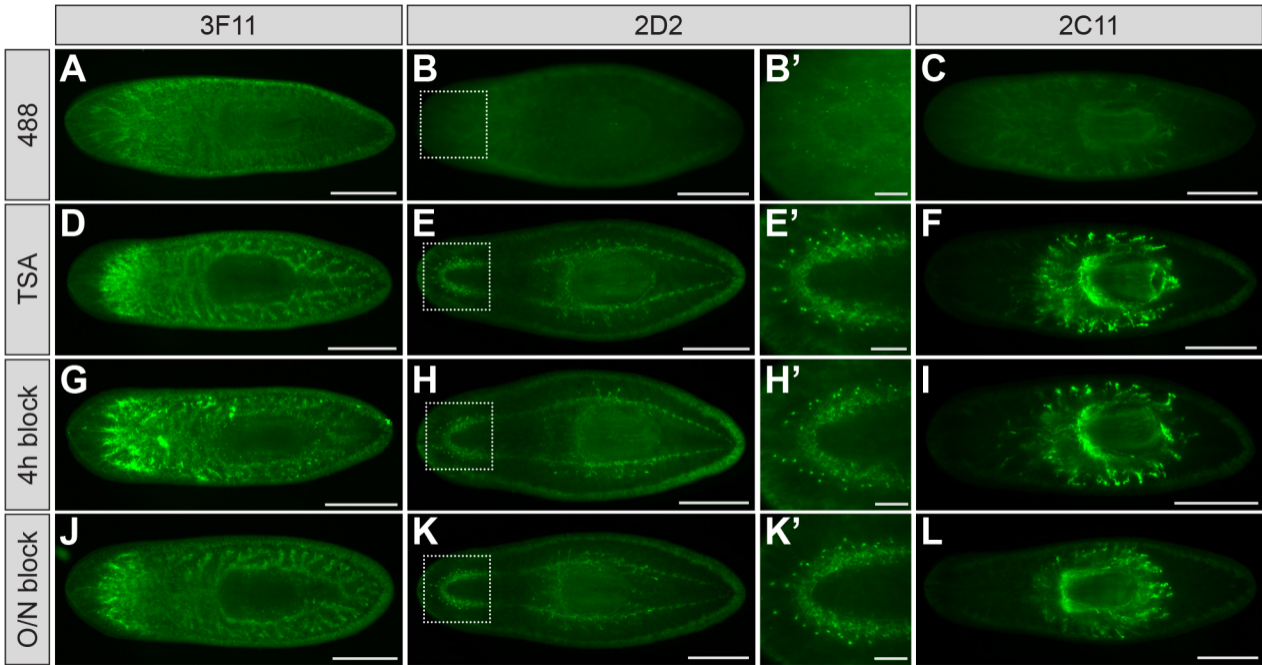

Supplement: Additional file 3: — Comparison of direct to indirect detection and effects of blocking duration on immunofluorescent labeling. (A-C) mAb detection using DyLight 488-conjugated secondary antibody. (D-F) mAb detection using HRP-conjugated secondary and FITC-tyramide (tyramide signal amplification). (G-I) mAb labeling after blocking for 4 hr prior to immunolabeling. (J-L) mAb labeling after blocking overnight (16-20 hr) prior to immunolabeling. For 3F11, animals were relaxed in magnesium, treated with 2% HCl, fixed in formaldehyde, and bleached in PBS. For 2C11 and 2D2, animals were processed identically except mucus was removed with 7.5% NAc and animals were bleached in methanol. For TSA vs. fluorophore-conjugated secondary comparison, animals were blocked overnight. For blocking comparison, TSA was used for detection. mAbs are indicated at the top of the figure. Scale bars: 100 μm (B’, E’, H’, K’); 500 μm (all other panels). [file 12861_2014_45_MOESM3_ESM.pdf]

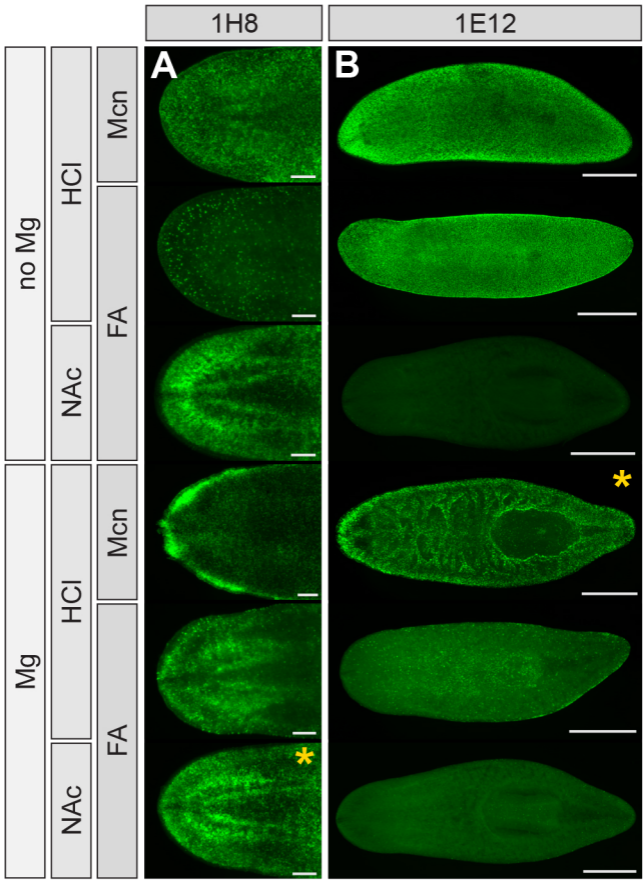

Supplement: Additional file 4: — Optimization of additional mAbs. (A) mAb 1H8 labeling is enriched in central nervous system nuclei, especially in NAc-treated samples. (B) mAb 1E12 labels nuclei. Signal is most robust after methacarn fixation; in combination with magnesium relaxation, epidermal signal is dramatically reduced, revealing intestinal nuclear labeling. Sample treatment parameters are indicated at left. In all panels, anterior is to the left. Yellow asterisks indicate conditions that yielded the most specific signal with minimal background labeling. All samples were bleached in methanol (16-20h). Scale bars: 100 μm (A); 500 μm (B). [file 12861_2014_45_MOESM4_ESM.pdf]

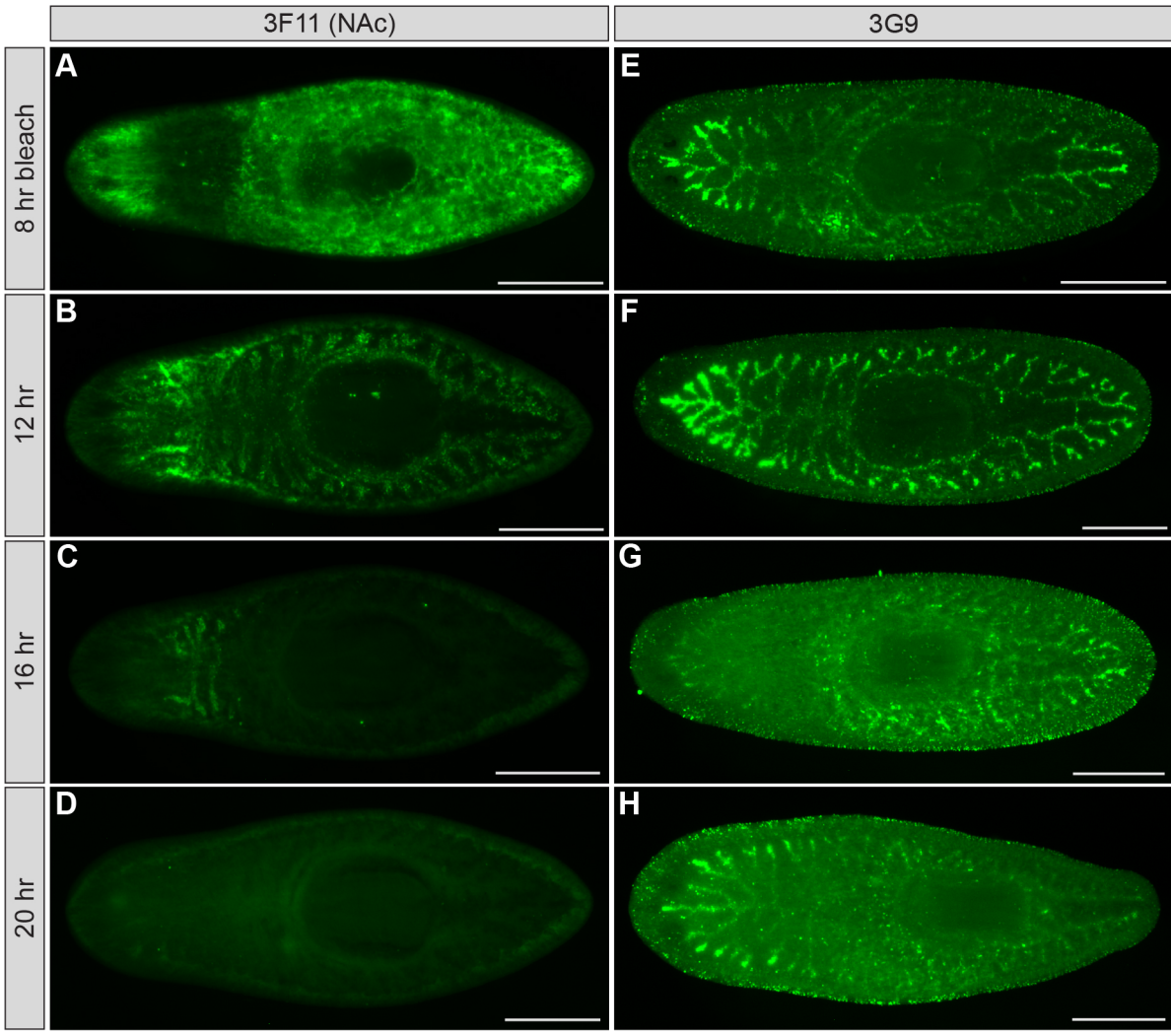

Supplement: Additional file 5: — Excessive bleaching adversely affects mAb labeling. (A-D) NAc-treated planarians labeled with mAb 3F11 after bleaching for the times indicated. (E-H) Planarians labeled with mAb 3G9 after bleaching for the times indicated. Planarians were relaxed in magnesium chloride, treated with 7.5% NAc (3F11) or 2% HCl (3G9), fixed in formaldehyde/Triton X-100 (3F11) or methacarn (3G9), and bleached in 6% H2O2/PBS (3F11) or 6% H2O2/methanol (3G9). Scale bars: 500 μm. [file 12861_2014_45_MOESM5_ESM.pdf]

**A**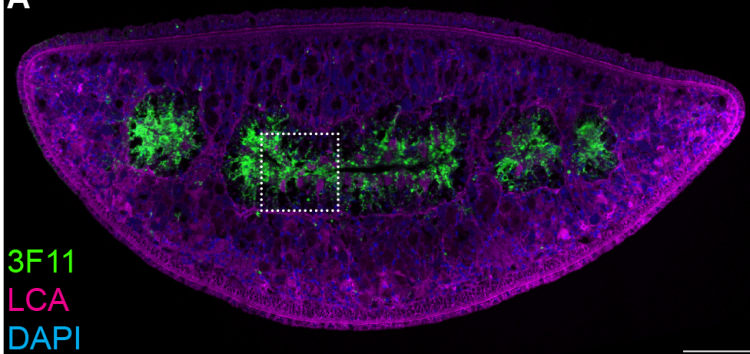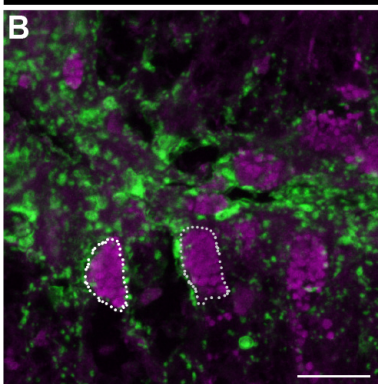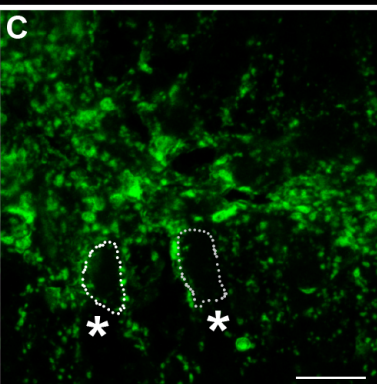**D**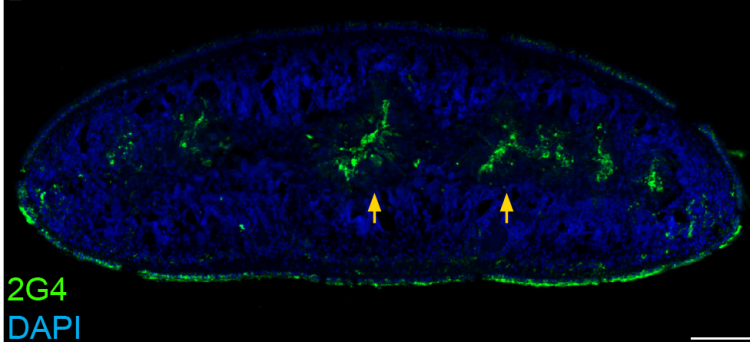**E**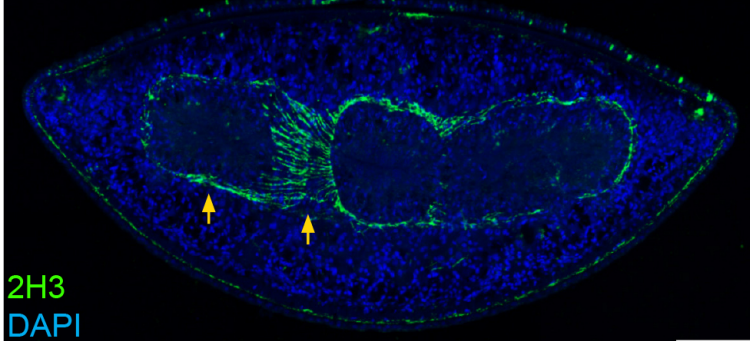

Supplement: Additional file 6: — mAb labeling of cryosections. (A) Cryosection labeled with mAb 3F11, Rhodamine-LCA, and DAPI. (B-C) High magnification of the region boxed in (A) shows the minimal overlap in labeling between 3F11 (phagocytes) and LCA (goblet cells). Asterisks indicate examples of 3F11-negative goblet cells. (D) 2G4 labeling of the apical region of the intestine (arrows) and ventral epidermis. (E) 2H3 labeling of muscle fibers around the intestine (arrows). 2H3 also weakly labels ventral body wall muscles under these conditions. 3F11- and 2G4-labeled samples were HCl-treated, FA-fixed, without AR. 2H3-labeled samples were HCl-treated, FA-fixed, and AR-treated. (A, D, E) Confocal projections of 20 μm thick cryosections. (B, C) 2 μm optical sections of the region boxed in (A). Scale bars: 100 μm (A, D, E); 20 μm (B, C). [file 12861_2014_45_MOESM6_ESM.pdf]

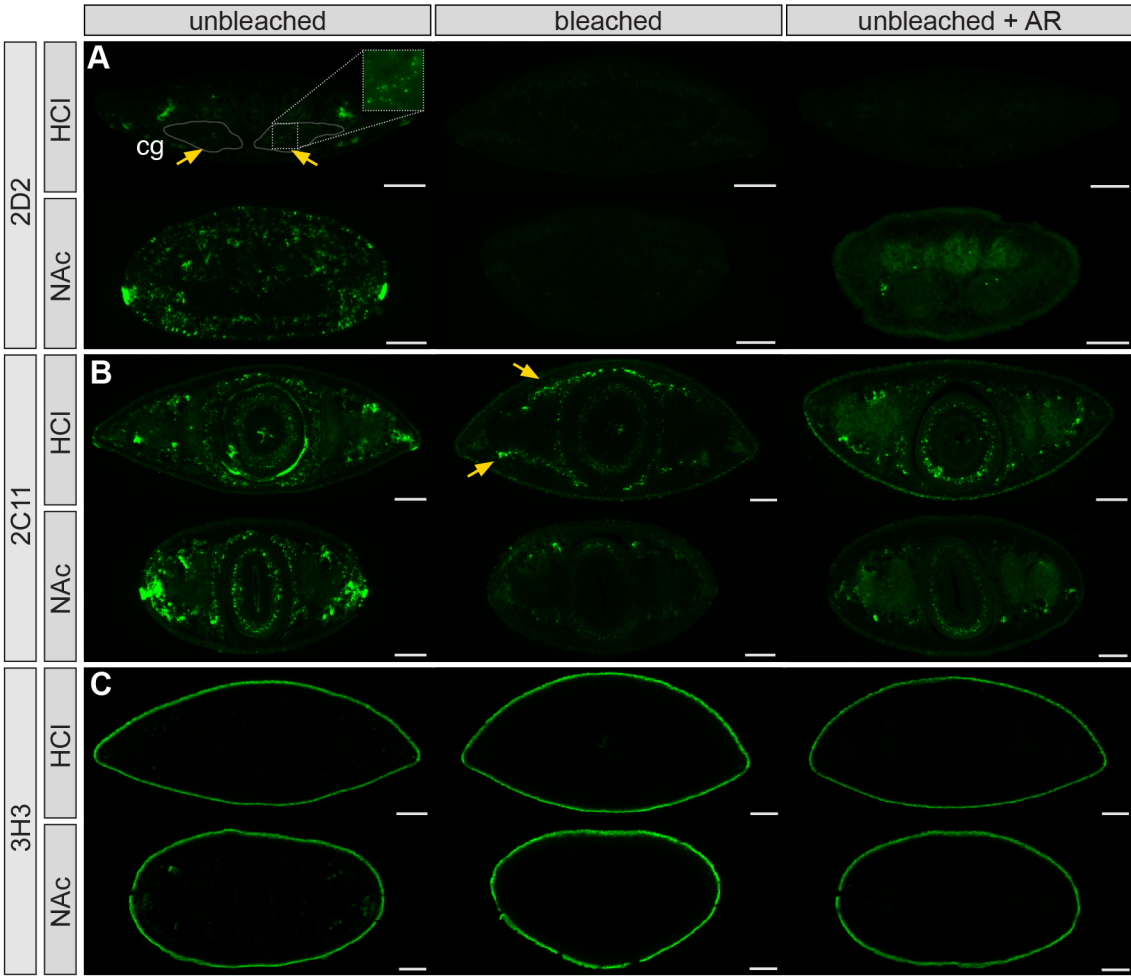

Supplement: Additional file 7: — mAb labeling of non-intestinal tissues on histological sections. Cryosections (20 μm) from formaldehyde-fixed planarians prepared as indicated and labeled with the antibodies shown. (A) Sections through the planarian brain labeled with mAb 2D2. Arrows indicate neuronal projections within cephalic ganglia (cg, dotted lines, and inset) in sections from HCl-treated, unbleached animals. (B) mAb 2C11 labels peripharyngeal secretory cells and their projections (arrows) in sections through the pharyngeal region. (C) mAb 3H3 labels epidermis. For all images, planarians were treated with HCl or NAc, and fixed in formaldehyde/Triton X-100 (20 min). Bleaching in 6% H2O2/PBS (12 hr) (middle column) was conducted prior to cryosectioning. Antigen retrieval was conducted after sectioning, on-slide, prior to immunolabeling. Dorsal is to the top in all images. Scale bars: 100 μm. [file 12861_2014_45_MOESM7_ESM.pdf]
